# Systematic review of outcome domains and instruments used in designs of clinical trials for interventions that seek to restore bilateral and binaural hearing in adults with unilateral severe to profound sensorineural hearing loss (‘single-sided deafness’)

**DOI:** 10.1186/s13063-021-05160-5

**Published:** 2021-03-20

**Authors:** Roulla Katiri, Deborah A. Hall, Catherine F. Killan, Sandra Smith, Pattarawadee Prayuenyong, Pádraig T. Kitterick

**Affiliations:** 1 National Institute for Health Research (NIHR) Nottingham Biomedical Research Centre (BRC), Ropewalk House, 113 The Ropewalk, Nottingham, NG1 5DU UK; 2 Department of Audiology, Mater Misericordiae University Hospital, Dublin, D07 R2WY Ireland; 3 Hearing Sciences, Division of Clinical Neuroscience, School of Medicine, University of Nottingham, Nottingham, NG7 2UH UK; 4 University of Nottingham Malaysia, Jalan Broga, 43500 Semenyih, Selangor Darul Ehsan Malaysia; 5 Yorkshire Auditory Implant Service, Bradford Teaching Hospitals Foundation NHS Trust, Bradford Royal Infirmary, Duckworth Lane, Bradford, BD9 6RJ UK; 6 Department of Otorhinolaryngology, Head and Neck Surgery, Faculty of Medicine, Prince of Songkla University, Songkhla, Thailand; 7 Nottingham University Hospitals NHS Trust, Queen’s Medical Centre, Derby Road, Nottingham, NG7 2UH UK

**Keywords:** Single-sided deafness, Outcome domains, Measurement instruments, Hearing interventions, Clinical trial design

## Abstract

**Background:**

This systematic review aimed to identify, compare and contrast outcome domains and outcome instruments reported in studies investigating interventions that seek to restore bilateral (two-sided) and/or binaural (both ears) hearing in adults with single-sided deafness (SSD). Findings can inform the development of evidence-based guidance to facilitate design decisions for confirmatory trials.

**Methods:**

Records were identified by searching MEDLINE, EMBASE, PubMed, CINAHL, ClinicalTrials.gov, ISRCTN, CENTRAL, WHO ICTRP and the NIHR UK clinical trials gateway. The search included records published from 1946 to March 2020. Included studies were those as follows: (a) recruiting adults aged 18 years or older diagnosed with SSD of average threshold severity worse than 70 dB HL in the worse-hearing ear and normal (or near-normal) hearing in the better-hearing ear, (b) evaluating interventions to restore bilateral and/or binaural hearing and (c) enrolling those adults in a controlled trial, before-and-after study or cross-over study. Studies that fell just short of the participant eligibility criteria were included in a separate sensitivity analysis.

**Results:**

Ninety-six studies were included (72 full inclusion, 24 sensitivity analysis). For fully included studies, 37 exclusively evaluated interventions to re-establish bilateral hearing and 29 exclusively evaluated interventions to restore binaural hearing. Overall, 520 outcome domains were identified (350 primary and 170 secondary). Speech-related outcome domains were the most common (74% of studies), followed by spatial-related domains (60% of studies). A total of 344 unique outcome instruments were reported. Speech-related outcome domains were measured by 73 different instruments and spatial-related domains by 43 different instruments. There was considerable variability in duration of follow-up, ranging from acute (baseline) testing to 10 years after the intervention. The sensitivity analysis identified no additional outcome domains.

**Conclusions:**

This review identified large variability in the reporting of outcome domains and instruments in studies evaluating the therapeutic benefits and harms of SSD interventions. Reports frequently omitted information on what domains the study intended to assess, and on what instruments were used to measure which domains.

**Trial registration:**

The systematic review protocol is registered on PROSPERO (International Prospective Register of Systematic Reviews): Registration Number CRD42018084274. Registered on 13 March 2018, last revised on 7th of May 2019.

**Supplementary Information:**

The online version contains supplementary material available at 10.1186/s13063-021-05160-5.

## Background

Single-sided deafness (SSD) arises when there is normal or near-normal hearing in one ear and a severe to profound sensorineural hearing impairment in the other ear [1]. The cause can be congenital [2, 3], sudden (e.g. Meniere’s disease [4], idiopathic [5, 6], due to autoimmune systemic diseases [7]); or progressive (e.g. vestibular schwannoma [8–10]). Regardless, SSD can lead to functional [11–16], psychological and social consequences [17–19].

The most commonly used treatments for SSD enable access to sounds on both sides of the head (‘bilateral’ hearing) by *rerouting* sounds from the impaired ear to the hearing ear [20, 21]. This effect can be achieved with a CROS (Contralateral Routing Of Signals) aid [20, 22–27] or a bone-anchored hearing aid (BAHA) [28–43] as well as other bone conduction devices like the ADHEAR [44, 45] or SoundBite™ [34, 46–49]. Alternatively, auditory input to the deaf ear can be *restored* (‘binaural’ hearing) by delivering sounds directly to the auditory pathway on the side of the impaired ear using auditory prostheses like a middle ear implant (MEI) [50, 51] or a cochlear implant [52–73].

Understanding which of these intervention approaches are optimal for patients with SSD should be based on robust evidence from well-designed trials [74, 75]. Certainly, with respect to health-related quality of life, there is a known inconsistency in the choice of measurement instruments in trials assessing the benefits of SSD interventions [76]. This inconsistency hinders comparison and meta-analysis across studies. The evidence synthesis in systematic reviews for example can be reliably conducted only if trials assess the same outcomes and measure them in the same way [77].

The challenge of synthesising evidence from trials and the importance of utilising valid instruments that effectively measure the intended audiological outcomes has been highlighted by Hall et al. [78]. Trialists should ideally base their choice of outcome measures on what is important and relevant to people making decisions about healthcare [79–81]. The question of what outcome domains are important and relevant to individuals with SSD when deciding whether an intervention works has yet to be asked in a systematic way. One attempt was made in 2017, but this was based on two discussions among professional experts in cochlear implantation at international conferences [1] and was intended for adoption in clinical practice. There has been no rigorous scrutiny of outcome reporting for rerouting *and* restoring interventions, no systematic patient involvement and no specific consideration of what should be recommended for clinical trials. As a consequence, investigators adopt markedly different study designs when assessing the clinical benefit of rerouting and restoring interventions for SSD.

The Core Rehabilitation Outcome Set for Single Sided Deafness (CROSSSD) study seeks to examine and address problems with inconsistent outcome reporting in SSD intervention trials. To achieve this, CROSSSD is developing a Core Outcome Set (COS) through a rigorous evidence-based process and by actively involving all relevant stakeholders in decision making. A COS is an agreed minimum set of outcomes or outcome measures which comprises a standardised collection of outcome domains that should be measured and reported worldwide, *as a minimum*, in all controlled trials within a research area [82–85]. Our published protocol [86] describes the process and this systematic review formed one of the first steps.

### Objectives

The primary objective of this systematic review was to identify those outcome domains and outcome instruments reported in published clinical studies evaluating *rerouting* and/or *restoring* interventions in adults with SSD. This information will be used to subsequently generate a ‘long list’ of candidate outcomes to be rated by SSD stakeholders according to whether each is important and critical to determine if an intervention works in this clinical population as part of the development of a Core Outcome Set (COS) [86].

There were two additional (secondary) objectives. One was to compare and contrast outcome domains and instruments reported for interventions that aim to re-establish (i) bilateral hearing (i.e. CROS aid, BAHA, ADHEAR, SoundBite™) and (ii) binaural hearing (i.e. MEI, CI). The other was to examine what outcome domains had been assessed and outcome instruments used as a function of time point after intervention. This information can be used to distinguish short- and long-term treatment-related changes. An exploratory objective was added to examine how the long list of candidate outcomes would be affected if the audiometric inclusion criteria were more lenient than our working definition adopted from the Van de Heyning et al. [1] consensus paper.

## Methods

### Searches

Details of the specific review questions, search strategy, study eligibility criteria, information sources, selection and data collection processes, quality assessment and data synthesis methods were published on PROSPERO international prospective register of systematic reviews in advance of data extraction [87]. There were no modifications to this PROSPERO protocol, but the Preferred Reporting Items for Systematic reviews and Meta-Analyses (PRISMA) statement [88] was modified for reporting purposes here. A populated PRISMA (2009) checklist can found in Additional file 1.

### Study inclusion and exclusion criteria

The eligibility was defined according to PICOS (Participant, Intervention(s), Comparator(s), Outcome, Setting) criteria. All included records assessed adults (male or female) aged 18 years or older with a diagnosis of congenital or acquired SSD. For the primary objective, diagnoses had to meet an audiometric profile independently defined through consensus [1]. For the worse-hearing ear, this required a threshold severity worse than 70 dB HL at audiometric frequencies ranging from 1 to 4 kHz, and for the better-hearing ear, this required a pure tone average of ≤ 30 dB HL across the same frequency range.

Eligible interventions comprised any medical devices designed specifically to restore bilateral (two-sided) or binaural (both ears) hearing. Any comparators in the study design were allowed, but studies exclusively evaluating other audiological interventions such as conventional hearing aids, assistive listening devices, audiological counselling, communication strategies or providing no intervention (unaided or placebo) were excluded. There were no restrictions on outcomes or research settings.

The systematic review included records reporting randomised controlled trials, quasi-randomised controlled trials, non-randomised controlled trials, before-and-after studies, cross-over studies, trial registrations and published protocols of such ongoing studies, and systematic reviews. Relevant systematic reviews were not subjected to the data collection process itself but were read to ensure all eligible records had been captured. Case control studies, cohort studies, non-systematic literature reviews (e.g. scoping reviews), practice guidelines, expert opinions, case series, case reports, book chapters, conference papers, manufacturers’ articles (e.g. white papers), animal studies and studies that use predictive modelling (e.g. prognostic factors established by acoustic test box measurements or studies performed with cadavers) were excluded. Original searches were performed from 1946, or the start date of databases, whichever was earlier, up to April 2018 inclusive. The searches were updated to 18th of March 2020. There were no restrictions on language of the publication.

During the data collection process, a small number of records were identified where information about age-related eligibility, audiometric thresholds or type of hearing loss in either the better or poorer hearing ear were missing. The corresponding author was contacted for more details by email, and a decision was made regarding inclusion in light of the new information provided. In cases where the author did not respond, an executive decision was taken to (i) include, (ii) exclude or (iii) use for sensitivity analysis; following discussion with one of the two senior authors (PTK or DAH). Cases in the sensitivity analysis were those trials or studies in which (i) participants’ audiometric profiles were close to our adopted SSD definition but differed from those criteria by up to 20 dB in individual frequencies either in the better or worse ear; (ii) the corresponding author was asked to clarify the audiometric profiles of participants but did not respond; and (iii) ongoing studies recruiting a mixture of participants (including children aged less than 18 years of age) and where it was not clear if results would be reported separately for the adults (aged 18 years or over).

### Information sources

Records were identified by searching electronic databases of research literature including (Table 1). Published, unpublished and ongoing studies were identified by electronically searching the following databases from their inception: EMBASE, MEDLINE, PubMed, CINAHL, ClinicalTrials.gov, ISRCTN, CENTRAL, ICTRP and the NIHR UK Clinical Trials Gateway. Electronic searchers were run by RK and PTK in March and April 2018 and then updated on 18th March 2020. In addition, a hand-search was conducted when reviewing the 76 published articles that had met the eligibility criteria at the abstract and full-text screening stages. Two potential articles were identified [27, 89], but following closer scrutiny neither met eligibility.

**Table 1:** Table summarising the electronic information sources used and the number of records identified

***Type of electronic search***	***Database***	***Date range***	***Number of items (n)***
***Academic databases***	Excerpta Medica dataBASE (EMBASE) via OvidSP	1974 to 18th March 2020	1463
	Medical Literature Analysis and Retrieval System Online (MEDLINE) via OvidSP	1974 to 18th March 2020	1144
	PubMed National Centre for Biotechnology Information	1946 to 18th March 2020	1223
	Cumulative Index of Nursing and Allied Health Literature (CINAHL) via EBSCO	1982 to 18th March 2020	384
		**Searched on**	
***Clinical trial registers and/or other sources***	ClinicalTrials.gov (www.clinicaltrials.gov)	18th March 2020	193
	International Standard Randomised Controlled Trials Number (ISRCTN) Registry (www.isrctn.com)	18th March 2020	48
	Cochrane Central Register of Controlled Trials (CENTRAL)	18th March 2020	962
	World Health Organization (WHO) International Clinical Trials Registry Platform (ICTRP) (www.who.int/ictrp)	18th March 2020	270
	NIHR UK Clinical Trials Gateway (www.ukctg.nihr.ac.uk)	18th March 2020	67

### Search strategy

The search strategy used in this systematic review was registered on PROSPERO [87]. Search terms for the PubMed, EMBASE and MEDLINE databases were informed by the PICOS criteria and comprised a set of terms to identify the population combined with a set of terms to identify relevant interventions. Where possible using the database interface, the scope of the search was limited to humans (not animals) and adults (not paediatric). An example of the search syntax for MEDLINE and EMBASE via OvidSP can be found in Additional file 2. The search strategy for the other databases was modelled on this search strategy and adapted where necessary to ensure the strategies were highly sensitive across each of the database interfaces. As an example, the syntax for search of the CENTRAL trials registry of the Cochrane Collaboration can be found in Additional file 3.

### Data management

RK, DAH and PTK were responsible for data management and maintained editorial rights. All identified records were saved into a Microsoft Excel Masterfile where records were tracked through the screening and data collection process by a unique study identification code.

### Selection process

All records identified by all database searches were uploaded into the EndNote software (Version X7) that was used to remove duplicates using the records’ title, list of authors, year of publication and journal of publication. In a few isolated cases, the abstract was also used to double-check if there was duplication, mainly for records that were published in a different language and the translated title or name of journal were different. The resulting number of records were subjected to eligibility screening.

Eligibility screening was carried out by RK, DAH, CFK, SS, PP and PTK, according to the published protocol [87]. For each record, the title and abstract screening decision was captured using a simple set of descriptors: include; unsure possibly include; exclude out of scope; exclude not SSD; exclude not adults; exclude wrong intervention; exclude wrong trial design; incomplete reference; abstract not accessible and sensitivity analysis (Additional file 4). Two co-authors (RK and DAH or PTK) independently performed and/or reviewed each step (i.e. title and abstract screening, full-text screening, data extraction, risk of bias assessment). Records that were included to conduct the sensitivity analysis only were extracted by RK alone. On rare occasions where agreement could not be reached between co-authors, disagreements were resolved by a third reviewer (DAH or PTK). The risk of bias assessment did not affect which findings were included in the analyses.

To enhance our data quality, data collection was guided by a data extraction protocol (Additional file 5), which informed the headings of the data masterfile. A calibration exercise was conducted for ten included records and reviewed for consistency across two coders, and the data extraction protocol was revised according to the lessons learned. No coder was permitted to be an author on any of the included records.

### Data items

Data items included PICOS fields as described by the PROSPERO record [87]. Participant data items relating to the inclusion criteria for each record were as follows: (1) SSD cause / aetiology, (2) age range, (3) mean age, (4) age standard deviation and (5) time since SSD diagnosis. Intervention data items recorded were as follows: (6) type of intervention device used and (7) time of implementation of intervention (how long after the onset of SSD the intervention was implemented). Data items describing the trial design included (8) the comparator device (if applicable), (9) the type of trial design and (10) the time duration for which each intervention or comparator device were used.

Outcome data items were as follows: (11) the outcome domain(s) specified by the investigators, (12) instruments specified by the investigators and (13) measurement time frame. Information relating to these three data items was recorded separately for all primary and secondary outcomes. Where authors were not explicit about the distinction between primary and secondary outcomes, the ‘Methods’ and ‘Results’ sections of each article were examined to identify any relevant information related to this distinction. If the study investigators did not explicitly distinguish multiple outcome domains as primary or secondary, they were all classified as primary.

Supplementary information was also extracted from each individual record on the following: (1) countries where the study was conducted, (2) corresponding author contact details, (3) source title (e.g. journal), (4) date of publication, (5) primary and/or secondary objective(s), (6) sample size, (7) description of any modifications to the study, particularly any discrepancies between the trial protocol and the subsequent report of the findings, and (8) any conflicts of interest identified by the authors. For (4), the date of publication recorded the date of the print copy, not the date of first submission, acceptance or the date of ‘online first’ publication. For (6), the estimated sample size was recorded for ongoing clinical trials where enrolment was not yet available. If any information was not reported, then ‘not stated’ was recorded in the corresponding field.

Where trial records had been consolidated into a single study, the data items reported in the synthesis related to the most recent study publication. For those records in which several pieces of information were consolidated for a single study, any inconsistencies between the protocol and the final reported study findings were noted (e.g. if the intended participant sample size was different in the published clinical trial record in comparison to the final study findings publication).

### Outcomes and prioritisation

The primary research question was to identify those outcome domains and outcome instruments reported in studies investigating interventions that seek to restore hearing in adults with SSD. There are no validated taxonomies specific to the hearing field and so for our classification of outcome domains, we chose to use the Dodd et al. [90] taxonomy. Strengths of this taxonomy are that it has been developed specifically for trial outcomes, is comprehensive, is not disease specific, is not limited to patient-centered outcomes and is applicable to trials irrespective of the field being studied. It comprises 38 categories across five core areas; death, physiological or clinical, life impact, resource use and adverse events. Classification of the review findings with respect to this taxonomy was conducted by RK and PTK. Finer breakdown of outcomes with the category ‘Ear and labyrinth outcomes’ was informed by a 2-day outcome domain grouping workshop that took place in July 2019 with members of the research steering group and the two public research partners. Details on the individual outcome domains review, consolidation and categorisation during the workshop can be found in the CROSSSD study protocol [86].

### Risk of bias

Given that the primary objective of this systematic review concerns methodology (not therapeutic effects), we limited the assessment of risk of bias to the data collection methods for consolidated records rather than any analysis of the intervention-related changes. The consolidated record data (e.g. outcome descriptors, published primary / secondary findings) was critically analysed for consistency of outcome reporting by two independent reviewers (RK and DAH). Risk of bias was assessed by analysing the reporting of outcomes both within and across manuscripts reporting study findings. Bias was determined by whether outcomes were reported prospectively through trial registration or published protocol, and whether outcomes were reported consistently between protocol / registration and study report. If consensus could not be reached on whether outcomes had been reported consistently, then disagreements were resolved by discussion with a third reviewer (PTK). No contact was made with corresponding authors to investigate the rationale of altered reporting. The quality of a record did not affect its inclusion in the synthesis of outcomes.

## Results

### Search results

**Fig. 1:**
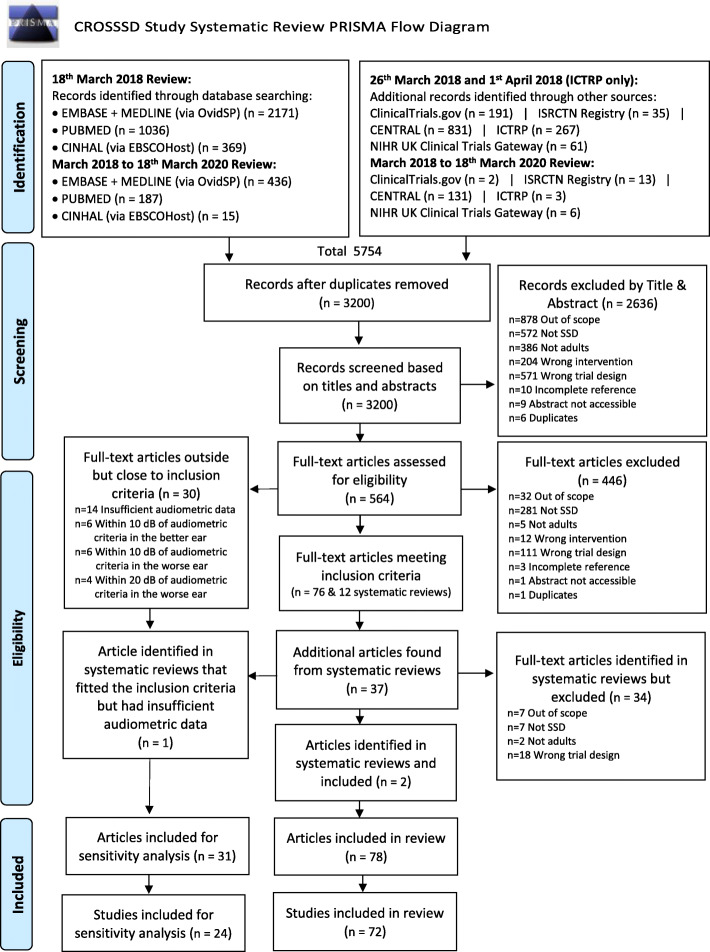
CROSSSD Reporting Items for Systematic Reviews and Meta-Analyses (PRISMA) flow diagram [91]

Figure 1 displays the results of the search strategy used to identify the relevant articles as recommended by the PRISMA statement [91]. The search strategy yielded 5754 records from which 2554 were excluded as duplicates. This resulted in a total of 3200 unique records being subjected to eligibility screening. Most exclusions during title and abstract eligibility screening were due to the study being out of scope (e.g. examined surgical methods, assistive hearing devices or hearing therapy techniques), the wrong trial design (e.g. case series, scoping reviews), not recruiting SSD participants or not recruiting adults, leaving 564 records (Fig. 1). For these, full texts were obtained and where necessary were translated to the English language. Full-text eligibility screening enabled a further 446 records to be excluded, with most exclusions due to participants not meeting the working definition of SSD [1] (*n* = 281) or using an ineligible trial design (*n* = 111). These exclusions left 118 records for data extraction, detailed as follows.

Full-text screening confirmed that 76 records reported trials in which the diagnosis of SSD fully met our criteria according to the Van de Heyning et al. [1] definition. The remaining 30 records reported participant criteria that narrowly missed the inclusion criteria (see eligibility criteria section above for details) but were sufficiently close to the criteria for inclusion in the sensitivity analysis. The 12 systematic review articles [76, 92–102] identified by the searches were reviewed to check for any overlooked trials or studies for inclusion, and 37 additional articles were identified by this approach. Full-text screening established that two met the inclusion criteria [72, 73], and one was included in the sensitivity analysis [25]. The rest were excluded due to wrong trial design (*n* = 18), the majority were conference papers, or they were out of scope (*n* = 7) [103–109], did not fit the audiometric criteria (*n* = 7) [110–116], or included children in their studies that were not reported separately from adults (*n* = 2) [117, 118]. References for the included records are given in Additional file 6 and the data masterfile containing information on all the data items is available for download (Additional file 7).

A number of records were consolidated for the purpose of reporting because they described the same trial or study. Two records by Härkönen et al. [53, 54] reported different measures obtained from the same group of participants. Two records reporting on a United Sates of America (USA) multicentre study [31, 32] were also consolidated as they reported on the same subset of participants. Four records [46–48, 119] reported on the same USA trial and participants, but presented different outcomes at different time scales so they were grouped. A French clinical trial registration (NCT02204618) by Marx et al. [120] was consolidated with the study findings record [69]. Similarly, a Swiss clinical trial registration (NCT01749592) [121] was consolidated with the published study findings record [66], a USA clinical trial (NCT02259192) [122] with the equivalent published record [65] and another French clinical trial (NCT02966366) [123] with its published record [67]. Two records by the Arndt et al. group reported on the same 11 participants but presented outcomes at 6 months [59] and at 12 months [52], so they were consolidated. Conversely, one composite article [38] reported the methods and results of five separate trials. Data from this article were extracted as five distinct studies. This re-classification led to a final dataset of 78 records reporting 72 studies that met full eligibility, and 31 records reporting 24 studies for the sensitivity analysis (Fig. 1). Of the 72 studies included, 37 assessed *rerouting* interventions, 29 studies assessed *restoring* interventions and just 6 studies directly evaluated both types of interventions.

### Data collection process

During eligibility screening, corresponding authors for 17 records were contacted to ask for more detail on the participant audiometric eligibility criteria or the definition of SSD adopted (see Additional file 8). Six authors responded with new information that allowed the screeners to come to a decision to (i) include for data extraction and synthesis (*n* = 1) [124], (ii) exclude from data extraction (*n* = 2) [125, 126] or (iii) include for sensitivity analysis (*n* = 3) [123, 127, 128]. Three emails were undeliverable, for these we decided to exclude two records [129, 130] but include one record [131] which was a clinical trial intending to recruit participants with SSD. Six authors did not respond [99, 132–136], and so we decided to include all six for sensitivity analysis. Two authors responded but their responses did not adequately clarify the query; one record [137] was included for sensitivity analysis and one [138] was excluded from data extraction.

The most common SSD diagnoses were sudden idiopathic or unknown cause (*n* = 218, 41%) and vestibular schwannoma (*n* = 134, 25%). The majority of rerouting intervention studies were conducted in the USA (*n* = 25, 66%). Restoring intervention studies were conducted in the USA (*n* = 8, 36%), Belgium and Germany (*n* = 4, 19%). Studies recruited a median of 10.5 participants (mean = 25.3, range 3–160). Most multicentre studies (*n* = 7) were conducted to evaluate restoring interventions rather than rerouting interventions.

### Outcome domains

To address our first objective, we examined the outcome domains extracted data from the 72 included studies and classified them for reporting using the Dodd et al. [90] taxonomy. Overall, 350 primary and 170 secondary data items were categorised across 19 of the 38 taxonomy categories (Table 2). Just over half (55%) of the reported outcome domains were physiological or clinical outcomes in the ear and labyrinth category (194 primary and 90 secondary outcome domain data items). Within this category, the most common items were from speech-related domains (e.g. speech in noise and speech in quiet), spatial-related domains (e.g. localisation and spatial hearing), hearing thresholds and tinnitus loudness. Life impact was the next most frequently reported core area (33%; 120 primary and 50 secondary outcome domain data items). The most popular categories within life impact were delivery of care and quality of life. We observed that investigators sometimes reported multiple assessments of the same outcome domain within a study, and so the caveat to these findings is that these frequencies do somewhat over-estimate the proportion of included studies reporting the outcome domain. A complete list of all reported outcome domains can be found in Additional file 9. A total of 22 outcome domain data items (4%) could not be coded because they were not clearly defined by the authors. Overall, 73 unique outcome domains were reported across the 72 included studies, 55 primary and 18 secondary outcome domains (Table 2).

**Table 2:** Summary of primary and secondary outcome domains and data items across all 72 included studies, classified according to core areas and categories defined by Dodd et al. [90]. Percentage values less than 1% are not reported

Taxonomy core area	Taxonomy categories	Number of data items (primary outcome domains)	% of total number of primary outcome domains	Number of data items (secondary outcome domains)	% of total number of secondary outcome domains	Number of unique outcome domains reported as primary	Number of unique outcome domains reported as secondary only
**Death**	1: Mortality / survival	0	–	0	–	0	0
**Physiological or clinical**	6: Ear and labyrinth outcomes	194	55.4%	90	52.9%	15	3
	7: Eye outcomes	0	–	1	–	1	0
	9: General outcomes	9	2.6%	1	–	2	0
	17: Nervous system outcomes	3	–	3	1.8%	2	0
	21: Psychiatric outcomes	3	–	0	–	1	0
	23: Skin and subcutaneous tissue outcomes	2	–	0	–	1	0
**Life impact**	25: Physical functioning	2	–	5	3.3%	2	3
	26: Social functioning	1	–	3	2.9%	1	2
	27: Role functioning	1	–	1	–	1	0
	28: Emotional functioning / well-being	7	2.0%	5	3.3%	5	3
	29: Cognitive functioning	2	–	3	2.9%	1	2
	30: Global quality of life	55	15.7%	16	9.4%	7	0
	31: Perceived health status	1	–	–	–	1	0
	32a: Delivery of care—satisfaction / patient preference	51	14.6%	17	10.0%	10	2
	32b: Delivery of care—acceptability and availability	0	–	1	–	0	1
**Resource use**	34: Economic	2	–	2	1.2%	2	0
	35: Hospital	1	–	0	–	1	0
	37: Societal / carer burden	0	–	1	–	0	1
**Adverse events**	38: Adverse events / effects	11	3.1%	5	2.9%	3	0
**Cannot code**	0: Cannot code	5	1.4%	17	10.0%	N/A	N/A
	**Total**	**350**		**170**		**55**	18

### Outcome instruments

Our first objective also asked about the outcome instruments used to measure the domains. For reporting purposes, measurement instruments are summarised according to whether they were as follows: (1) investigator administered, (2) patient-reported outcome measures (PROMs) and (3) unclear or unknown (Table 3). Within each of these categories, a finer breakdown was performed that was relevant to the instrument category (e.g. PROMs could be a numerical rating scale, multi-item questionnaire or diary).

**Table 3:** Summary of the number of more common measurement methods used to assess treatment outcomes in each domain category from the Dodd et al. [90] taxonomy. Only items where there were more than 10 reports of the outcome domain (Additional file 9) and only those methods reported more than once across the 72 included studies are selected for reporting here. See Additional file 10 for a complete list of instruments

Outcome domains	Investigator administered				Patient-reported outcome measures (PROMs)			Unclear
	Psychophysical instruments	Objective instruments	Technical and lab measures	Investigator observation / judgement	Numerical rating scale	Multi-item questionnaire	Diary	Unclear
**Physiological or clinical core area, 6: ear and labyrinth outcomes**								
Hearing thresholds	8	1	–	–	–	–	–	–
Speech in noise	47	–	–	–	–	2	–	–
Speech in quiet	18	–	–	–	–	2	–	–
Speech hearing	6	–	–	–	–	3	–	–
Tinnitus loudness	1	–	–	–	2	–	–	–
Spatial hearing	1	–	–	–	–	3	–	–
Localisation	33	–	–	–	–	1	–	–
Quality of hearing	–	–	–	–	2	1	–	2
Reverberation	–	–	–	–	1	1	–	–
**Life impact core area, 30: global quality of life**								
Tinnitus symptom severity	–	–	–	–	–	9	–	–
Hearing disabilities	–	–	–	–	–	9	–	–
Disease-specific quality of life	–	–	–	–	–	6	–	–
**Life impact core area, 32a: delivery of care—satisfaction / patient preference**								
Device benefit	–	–	–	–	2	8	–	–
Device use	–	–	3	–	1	2	1	1
Satisfaction	–	–	–	–	2	5	1	–
Aversiveness	–	–	–	–	–	1	–	
**Adverse events core area, 38: adverse events / effects**								
Adverse effects	–	–	–	1	–	2	–	6

Collating information about the measurement instruments reported in the 72 studies revealed a large number of ways to measure the domains of interest and the fact that no single instrument was used by all studies. We observed that reporting was strongly biased towards benefits not harms. Counting the exact number of instruments is not straightforward because some of the instruments were reported both as global scores and subscale scores across different studies and different authors administered the same instrument to assess different outcome domains. For example, the Glasgow Hearing Aid Benefit Profile (GHABP) [139] was reported in various forms under hearing handicap, pre-intervention disability, device benefit, device use, satisfaction, and residual (aided) disability. Regarding the Abbreviated Profile of Hearing Aid Benefit (APHAB) [140], performance was most often reported as a subscale not a global score, including all three speech communication subscales (Ease of communication, Reverberation and Background noise subscales). For the purposes of reporting here, the different forms reported by the authors all contribute to the data item counts and so the numbers may over-estimate the number of instruments per se. For that reason, we refer to these data as measurement ‘methods’ not ‘instruments’. A summary of the number of methods used to measure the outcomes across the most popular Dodd’s taxonomy categories is given in Table 3. For transparency, a comprehensive listing of all methods can be found in Additional file 10, organised according to the domains within Dodd’s taxonomy categories.

Considering the ear and labyrinth outcome domains, the 18 outcome domains were assessed by 133 different measurement methods. A description of the more popular methods is given in Table 4. The most common approach was an investigator-administered psychophysical instrument. This was true for all of the speech-related domains (i.e. speech in noise, speech in quiet and speech hearing), spatial localisation and hearing thresholds. Speech performance was most often measured by a speech reception threshold, although there were many different testing methods. There was no clear preferred method for measuring speech in quiet, while speech in noise was most often assessed using the Hearing in Noise Test (HINT) [141]. However, even here the choice of background noise was not consistent across studies. Localisation performance was most often measured by localisation accuracy using a horizontal circular or semi-circular array of loudspeakers. However, again the number of loudspeakers and angular separation between sound sources varied across studies. Perhaps unsurprisingly, hearing thresholds were most often assessed using pure-tone audiometry which tends to have a more standardised testing method. Tinnitus loudness was commonly measured using a visual analogue scale (VAS), which is a form of PROM.

**Table 4:** Listing of all unique measurement methods used to assess the most common outcomes in Dodd’s taxonomy physiological or clinical #6, ear and labyrinth category. Only those domains where there was more than one report of the outcome measurement method are selected for reporting here. See Additional file 10 for full details

Measurement methods (***n*** > 1) split by outcome domains and type of method	Primary outcomes	Secondary outcomes
**Hearing thresholds, investigator administered**		
Psychophysical		
Pure-tone audiometry	10	–
Pure-tone audiometry (bone conduction only)	2	–
Soundfield audiometry	2	–
**Speech in noise, investigator administered**		
Psychophysical		
Bamford-Kowal-Bench Speech in Noise test (BKB-SiN) in four-talker babble, Speech Reception Thresholds (SRT)	3	–
Bamford-Kowal-Bench Speech in Noise test (BKB-SiN) in multi-talker babble, Speech Reception Thresholds (SRT)	2	–
Hearing In Noise Test (HINT) in multi-talker babble, Speech Reception Thresholds (SRT)	2	–
Hearing in Noise Test (HINT) in R-space restaurant noise, Speech Reception Thresholds (SRT)	2	–
Hearing in Noise Test (HINT) noise not specified, Speech Reception Thresholds (SRT)	2	–
Hochmair-Schulz-Moser sentence test in speech-shaped noise, Speech Reception Thresholds (SRT)	3	–
Leuven Intelligibility Sentences Test (LIST) noise not specified, Speech Reception Thresholds (SRT)	2	–
Oldenburg Sentence Test (OlSa) noise not specified, Speech Reception Thresholds (SRT)	5	–
Quick Speech-In-Noise (QuickSIN) test in multi-talker babble, Speech Reception Thresholds (SRT)	2	–
Speech Intelligibility In Noise (SPIN) test in multi-talker babble, percent correct	3	–
Speech-in-noise test in speech (not specified), Speech Reception Thresholds (SRT)	2	–
**Speech in noise, PROM**		
Multi-item questionnaire		
APHAB background noise subscale	5	9
**Speech in quiet, investigator administered**		
Psychophysical		
Consonant-Nucleus-Consonant (CNC) word list	3	–
Freiburger monosyllabic word discrimination in quiet, Speech Reception Thresholds (SRT)	2	–
Monosyllable test (67S test), Japanese version	2	
**Speech hearing, PROM**		
Multi-item questionnaire		
Abbreviated Profile of Hearing Aid Benefit (APHAB) ease of communication subscale	5	9
Speech, Spatial and Qualities of Hearing (SSQ) speech subscale	4	9
**Tinnitus loudness, investigator administered**		
Psychophysical		
Tinnitus Loudness Matching	1	3
**Tinnitus loudness, PROM**		
Numerical rating scale		
Numerical rating scale (not specified)	2	1
Visual analogue scale (not specified)	6	–
**Spatial hearing, PROM**		
Multi-item questionnaire		
Speech, Spatial and Qualities of Hearing (SSQ) spatial subscale	3	8
Spatial Hearing Questionnaire (SHQ)	2	–
Spatial Hearing Questionnaire (SHQ), various subscales	–	8
**Localisation, investigator administered**		
Psychophysical		
Horizontal semi-circular array of 7 loudspeakers, angular separation 30̟°, localisation accuracy	2	1
Horizontal circular array of 5 loudspeakers, angular separation 45°, localisation accuracy	2	–
Horizontal circular array of 9 out of 33 loudspeakers, angular separation 5.6°, localisation accuracy	1	1
Horizontal circular array of 19 loudspeakers, angular separation 10°, localisation accuracy	2	–
Localisation from one or multiple loudspeakers (not specified)	1	2

The seven global quality of life outcome domains were assessed by 36 different measurement methods. A description of the more popular methods is given in Table 5. The most common method of assessment was a PROM, in the form of a multi-item questionnaire. Most frequently reported were the Speech, Spatial and Qualities of Hearing (SSQ) [16], Glasgow Benefit Inventory (GBI), single-sided deafness (SSD) questionnaire and Tinnitus Handicap Inventory (THI) [142].

**Table 5:** Listing of all unique measurement methods used to assess the most common outcomes in the Dodd’s taxonomy life impact #30, global quality of life category [90]. Only those domains where there was more than 1 report of the outcome measurement instrument are selected for reporting here. See Additional file 10 for full details

Measurement methods (***n*** > 1) split by outcome domains and type of method		Primary outcomes	Secondary outcomes
**Tinnitus-symptom severity, PROM**			
Multi-item questionnaire	Tinnitus Handicap Inventory (THI)	7	1
	Tinnitus Questionnaire (TQ)	2	1
	Tinnitus Questionnaire (TQ), German version	3	-
	Tinnitus Reaction Questionnaire (TRQ)	3	-
**Hearing disabilities, PROM**			
Multi-item questionnaire	Speech, Spatial and Qualities of Hearing (SSQ)	8	-
**Disease-specific quality of life, PROM**			
Multi-item questionnaire	Bern Benefit in Single Sided Deafness (BBSS) questionnaire	1	1
	Glasgow Benefit Inventory (GBI)	3	2
	Nijmegen Cochlear Implant Questionnaire (NCIQ)	1	2
	Single Sided Deafness (SSD) questionnaire	4	-
	Multi-item, multi-domain questionnaire (author's own)	-	2

The 12 delivery of care (Satisfaction / patient preference) outcome domains were assessed by 37 different measurement methods. A description of the more popular methods is given in Table 6. Once again, the most common method of assessment was a PROM, in the form of a multi-item questionnaire. The most frequently reported were the Abbreviated Profile of Hearing Aid Benefit (APHAB) [140] and the Glasgow Hearing Aid Benefit Profile (GHABP).

**Table 6:** Listing of all unique measurement methods used to assess the most common outcomes in the Dodd’s taxonomy life impact #32a, Delivery of care (Satisfaction / patient preference) category. Only those domains where there was more than one report of the outcome measurement instrument are selected for reporting here. See Additional file 10 for full details

Measurement methods (***n*** > 1) split by outcome domains and type of method		Primary outcomes	Secondary outcomes
**Device benefit, PROM**			
Multi-item questionnaire	Abbreviated Profile of Hearing Aid Benefit (APHAB)	6	-
	Glasgow Hearing Aid Benefit Profile (GHABP)	4	-
	Multi-item, multi-domain Questionnaire (author's own)	3	-
Numerical rating scale	International Outcome Inventory for Hearing Aids (IOI-HA), single item on benefit	1	1
	Visual Analogue Scale (not specified)	1	-
**Device use, investigator administered**			
Technical and lab measures	Device log (not specified)	2	-
	Device log average usage (hrs / day)	3	-
**Device use, PROM**			
Multi-item questionnaire	Glasgow Hearing Aid Benefit Profile (GHABP), hearing aid use subscale	2	-
**Satisfaction, PROM**			
Multi-item questionnaire	Glasgow Hearing Aid Benefit Profile (GHABP), various subscales	1	2
	Multi-item, multi-domain questionnaire (author's own)	2	-
**Aversiveness, PROM**			
Multi-item questionnaire	Abbreviated Profile of Hearing Aid Benefit (APHAB) aversiveness subscale	5	6

### Comparing the report of outcome domains and instruments across interventions

One of our secondary objectives was to compare and contrast outcome domains and instruments reported for interventions that aim to re-establish (i) bilateral hearing (i.e. CROS aid, BAHA, ADHEAR, SoundBite™) through *rerouting* and (ii) binaural hearing (i.e. MEI, CI) through *restoring*.

Across the 72 included studies, 37 assessed *rerouting* interventions only and 29 assessed *restoring* interventions only. The remainder assessed both interventions in the same study design and so are not included in this comparison. Generally speaking, the two intervention approaches assessed the same outcome domains. But there were several notable exceptions. Tinnitus-related outcomes were almost exclusively limited to studies evaluating restoring interventions (reported 43 times) rather than rerouting interventions (reported once). The same was true for brain-related assessments of neural activity (restoring studies reported three times; rerouting studies none). In contrast, rerouting studies were also much more concerned about aversiveness (reported 10 times) than were restoring studies (reported once). Furthermore, all dental outcomes were limited to rerouting studies. In fact, all eight reports came from a single study evaluating the SoundBite™ intraoral device [46–48, 119]. A summary of the split across the interventions is given in Additional file 9.

Overall, restoring intervention studies reported a greater proportion of investigator-administered tests than PROMs, while rerouting intervention studies reported more of a balance of these two instrument types. It was not possible to determine the effect of intervention on the choice of measurement methods because the number of times each method used was generally very small. Perhaps the most striking effect observed was that speech hearing was assessed using the APHAB [140] ease of communication subscale in rerouting studies (reported 13 times) much more often than in restoring studies (reported just once).

### Use of measures over time frame

For both primary and secondary outcomes, there was significant variability in the duration of follow-up period, ranging from acute (baseline) testing to 10 years post-intervention. There was notable inconsistency in the number of testing sessions, from a single session to ongoing daily records [50], and when they were conducted after device fitting or surgery. For reporting, time frames were grouped into measures taken in a single session only, at a time point less than 3 months after baseline (‘acute’), at a time point from 3 months to less than 1 year after intervention (‘early’ acclimatisation) and at 1 year or more after intervention (‘long’). Eighteen of the 72 included studies were designed as a single session (12 rerouting, 5 restoring, 1 both), 26 had at least one acute follow-up (16 rerouting, 9 restoring, 1 both) mostly at 1 month after baseline, 31 had at least one early follow-up (11 rerouting, 16 restoring, 4 both) mostly at 6 months after baseline and 26 had at least one long follow-up (6 rerouting, 16 restoring, 4 both).

**Fig. 2:**
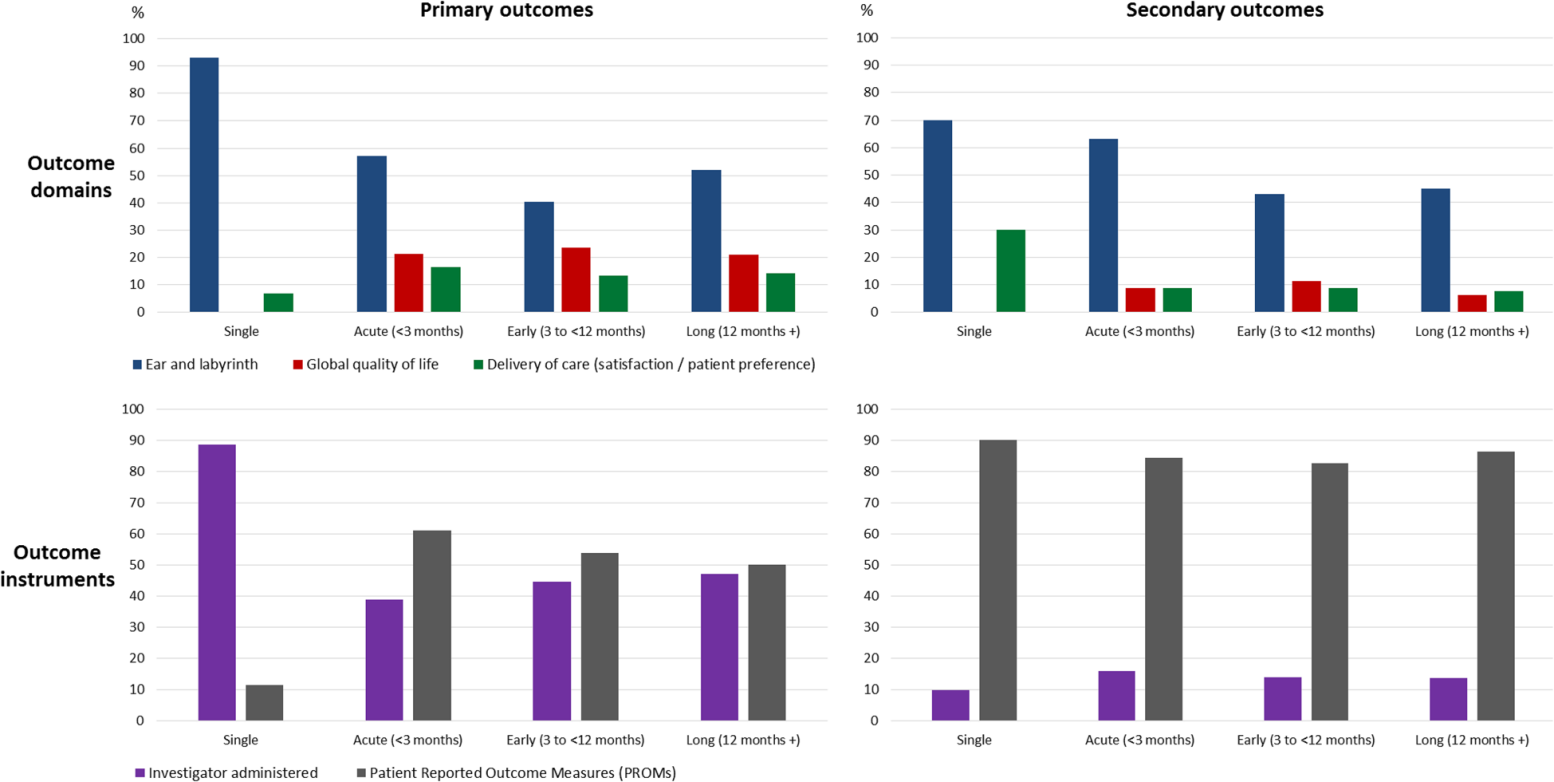
Top panels illustrate reporting of the three major outcome domain taxonomy categories over the successive follow-up time points. Bottom panels illustrate reporting of the two major measurement methods over time. All data are reported as a percentage, normalised to the total number of outcome data items assessed at that time point and calculated separately for primary and secondary outcomes

We evaluated whether there was any change over time in the choice of primary and secondary outcome domains and measurement methods by classifying the data according to the three major Dodd’s taxonomy categories [90] (#6 Ear and labyrinth, #30 global quality of life and #32a delivery of care), and according to whether they were investigator-administered tests or PROMs (Fig. 2).

Single-session studies almost exclusively focused on hearing-related outcome domains, but ear and labyrinth accounted for about 50% of the outcomes assessed, even at the longest time frame. Similarly, single-session studies almost exclusively used investigator-administered testing methods, but over time, a more 50/50 balanced was observed between these and PROMs. This pattern was true for the primary outcomes. However, secondary outcomes were almost always used PROMs, irrespective of the time frame.

### Sensitivity analysis

The final, exploratory objective examined whether we would identify any additional outcomes if we included studies where the audiometric eligibility criteria were more lenient than our working definition adopted from the Van de Heyning et al. [1] consensus paper. The outcome domains reported by the 24 additional studies were coded in the same way as described previously. Overall, 205 primary and 80 secondary data items were categorised. None of these reported outcomes had not already been captured by the 72 included studies.

### Risk of bias

Assessment of the 72 included studies focused on (a) whether the outcomes were reported prospectively, and (b) if yes, whether there was consistency between the prospective registration and the published study. Notes on conflicts of interest and study design were also taken.

Although there were 11 clinical trial registrations, only four studies with reported findings had been pre-registered. One assessed the SoundBite™ [46] and three assessed cochlear implantation [120, 121, 131]. The SoundBite™ study had two discrepancies: adverse effects were not reported in the protocol (NCT01933386) [131] but were reported in the published record discussion, and the pure-tone audiometry (PTA) thresholds range were planned up to 4 kHz in the protocol but reported up to 2 kHz in the study report. All three cochlear implant studies had discrepancies between the measures planned in the registered protocol and those actually reported in the study findings. Other discrepancies included lack of clarity in the report on whether adverse events were assessed at 1 month post-implantation, as per protocol (NCT02259192) [122] and differences between the planned and reported measurement time frames (NCT01749592) [121].

## Discussion

There is a growing general recognition that insufficient attention has been paid to the outcomes measured and reported in clinical trials. The CROSSSD study group has established the need for a core outcome set for SSD interventions and we are the first to identify existing knowledge about outcomes using a systematic review methodology.

### Principal findings

Most studies included in the review evaluated rerouting interventions rather than restoring interventions. There was a large variation in the reported outcome domains, with most studies concentrating on physiological or clinical outcomes, followed by life impact outcomes. Only a small minority of studies reported on resource use and adverse events. Investigators did not always report what their intended outcome domain was, suggesting that their chosen instruments were not actively matched to an outcome domain. With regard to instruments chosen by investigators, a large inconsistency was observed with investigator-administered tests mostly adopted, focusing mainly on speech in noise and spatial-related testing. A diversity within these categories of instruments was also observed with a plethora of signal and noise configurations that do not always fit existing recommendations that aim to reveal both the benefits and drawbacks of hearing devices. Similarly, multi-item questionnaires are frequently utilised but there is no consensus in their selection, nor the intended outcome domains to be measured. Although the range of functional difficulties imposed by SSD, as well as the impact on individual’s social and psychological well-being, are well documented [17], similar to other interventions in the hearing field, they are not always assessed in a systematic manner [78, 143, 144]. The time frame when interventions are assessed also varies, so it is challenging to compare the short- and long-term treatment-related changes for rerouting and restoring interventions.

### Comparison with other studies

Our review identifies limitations in the range of reported outcomes in clinical trials that are reflected more broadly across clinical practice in ENT and audiology. In 2016, Van de Heyning led a several expert panel discussions to reach a consensus on a clinical protocol for SSD including a minimum set of outcome measures [1]. This group recommended a core set of three ear and labyrinth and two life impact measures, tested using investigator-administered tests and PROMs, respectively. Ear and labyrinth measures were as follows: (i) hearing thresholds using pure-tone audiometry, (ii) speech in noise perception using a standard audiometric and validated sentence test and a free-field setup in a sound-treated room and (iii) sound localisation using a free-field system with at least seven loudspeakers horizontally distributed with equal angular separation, again in a sound-treated room. In our review, we observed that these were some of the most popular domains reported across the 72 included studies. Common speech in noise materials included the Hearing in Noise Test (HINT) sentences, Oldenburg sentence test (OlSa) and the Speech Intelligibility In Noise (SPIN) test. Measurements for sound localisation perhaps diverged the most from this expert panel recommendation, with numerous studies either using fewer speakers or testing front and back localisation in a circular array. Recommended life impact measures were as follows: (i) quality of life using both disease-specific (speech, spatial, and qualities of hearing (SSQ)) and generic health-related (Health Utilities Index (HUI) Mark 3) questionnaires and (ii) delivery of care using a measure of device use (data logging or patient report). In our review, we actually coded the SSQ questionnaire as an ear and labyrinth assessment (not life impact) because it was most often reported as separate subscale scores for speech hearing, spatial hearing and quality of hearing. We also observed that Health Utilities Index (HUI-3) [145] was rarely reported across the 72 included studies (others were EuroQol-5D-3L [146], WHOQOL-BREF [147] and SF-36 [148]).

Van de Heyning et al. [1] also recommended tinnitus assessment if applicable, namely tinnitus loudness (using a visual analogue scale (VAS)) and tinnitus symptom severity (using the Tinnitus Functional Index). Our review confirmed that these domains were often limited to restoring interventions (cochlear implant) studies and therefore were perhaps considered less relevant to rerouting interventions for SSD by investigators. It is possible that this recommendation reached consensus because the panel comprised cochlear implant experts attending a cochlear implant conference.

In terms of time frame for outcome measurement, Van de Heyning et al. [1] recommended that outcomes should be collected at baseline, and at 1, 3, 6 and 12 months after intervention. This would mean that all studies should span the acute, early and long-term time points coded in our review. Nevertheless, less than half of the included studies assessed outcome at these time points.

### Strengths and limitations of the study

Our review was guided by good practice as set out in the Core Outcome Measures in Effectiveness Trials (COMET) handbook [77]. In terms of outcome extraction from the academic literature, Williamson et al. [77] state ‘it is recommended that all are extracted verbatim from the source manuscript’. This posed some challenges when it came to coding the domains because different investigators used different terminology even when they were probably referring to the same construct. In order to collate the data, our team had to recode some of the domain names, conducting this task in pairs to reduce the impact of individual opinion. For transparency, we provide the authors’ original description and our recoding in Additional file 7. This allows external critical review of the CROSSSD core outcome set, whose development will be informed by this review, right back to its inception.

There is currently no consensus on how clinical trial outcomes should be classified [149]. COS researchers often simply agree ‘themes’ in discussion with advisory groups [150]. However, it is recognised that this lack of a standardised outcome classification system results in inconsistencies due to ambiguity and variation in how outcomes are described across different studies. Recently, a new taxonomy for outcome classification has been developed to promote efficient searching, reporting and classification of trial outcomes [90]. Strengths are that it focuses on general outcomes, is comprehensive, is not disease specific and is applicable to trials for any disease or health condition. Although not part of our published protocol [87], we felt that it was sufficiently important to implement this standard taxonomy in this systematic review. While our findings are presented using this taxonomy, it was insufficient to delineate the different outcome domains with the ear and labyrinth category and so we expanded this category using our own subcategories. Again for transparency, we provide full details of our customised sub-categorisation in Additional file 10.

Also recommended in the COMET handbook [77] is to perform the systematic review in stages to check if outcome saturation is reached. In this sense, our sensitivity analysis can be considered such a check. We had identified 24 additional studies that just missed the eligibility criteria for inclusion, but none of these studies reported any novel outcome domains that had not already been captured in the first stage. As Williamson et al. [77] state ‘If there are no further outcomes of importance then the systematic review may be considered complete’.

The longer-term intention of this work is to develop a core outcome set that identifies by consensus a minimum standard for reporting in clinical trials of SSD in adults. This review makes a specific contribution to that endeavour by identifying domains that have been defined in relevant clinical trial designs to date. We recognise that systematic reviews of outcomes simply aggregate the opinions of previous researchers on what outcomes they deemed important to measure. The outcome domains collated in this review will be put forward as potential candidates as outcome domains in a long list that will be considered by a range of stakeholders using a Delphi consensus method [77]. For that long list to be truly comprehensive, it is important to also give participants the option to nominate any new domains that they might consider missing. Here, the patient perspective might be important as they have not yet hitherto been actively involved in COS decision making.

## Conclusions

This review highlights outcome domains and instruments reported by studies that have evaluated rerouting and/or restoring interventions for SSD in adults. The extracted data provides a meticulous catalogue of investigators’ chosen outcome domains of which the majority were successfully categorised using the Dodd et al. [90] taxonomy outcome classification system. Our findings emphasise the need to improve trial design and reporting in this area of health research. We hope that guidelines that have been developed explicitly with both rerouting and restoring interventions in mind will have broader take up across the ENT and audiology communities. To improve reporting, we also draw attention to the specialised CONSORT guidelines for reporting harms-related issues in a randomised controlled trial [83].

## Supplementary Information

**Additional file 1.** PRISMA checklist. PRISMA checklist of items to include when reporting a systematic review.

**Additional file 2.** Search syntax – MEDLINE and EMBASE. Search syntax for Excerpta Medica dataBASE (EMBASE) and Medical Literature Analysis and Retrieval System Online (MEDLINE) via OvidSP.

**Additional file 3.** Search syntax - CENTRAL. Search syntax for Cochrane Central Register of Controlled Trials (CENTRAL).

**Additional file 4.** Title and abstract screening. Methodology for CROSSSD title and abstract article screening.

**Additional file 5.** Data extraction. CROSSSD data extraction protocol.

**Additional file 6. **References for all included records. List of records that explicitly fitted the systematic review inclusion criteria as defined by our PROSPERO protocol and were included in the systematic review data extraction (*n* = 78), those that closely fitted and were included for sensitivity analysis only (*n* = 31), and the 12 systematic reviews.

**Additional file 7.** Data masterfile. An editable version of the data masterfile comprising three worksheets for the 72 studies fitting all criteria, the 24 studies closely fitting the audiometric criteria and the 12 systematic reviews.

**Additional file 8.** Author queries. Table of records containing missing data that was queried to the corresponding author by email, and outcome.

**Additional file 9.** Complete list of reported outcome domains. A comprehensive list of all reported primary and secondary outcome domains reported across the 72 included studies, classified according to the Dodd et al. [90] taxonomy. Categories within each core area are arranged by the most frequently used first. The number of individual studies that reported each primary and secondary domain is also listed. N/A = none reported.

**Additional file 10. **Complete list of measurement instruments. A comprehensive list of all primary and secondary measurement instruments (*n* = 344) reported across the 72 included studies, classified according to our domain taxonomy. Where the name of a standard instrument is not given by the authors, a short description is provided instead.

## Data Availability

Upon the completion of the study, supporting data will be available upon request.

## Abbreviations

APHAB: Abbreviated Profile of Hearing Aid Benefit
BAHA: Bone-anchored hearing aid
BRC: Biomedical Research Centre
CENTRAL: Cochrane Central Register of Controlled Trials
CINAHL: Cumulative Index of Nursing and Allied Health Literature
COMET: Core Outcome Measures in Effectiveness Trials
COS: Core Outcome Set
CROS: Contralateral Routing Of Signals
CROSSSD: Core Rehabilitation Outcome Set for Single Sided Deafness
EMBASE: Excerpta Medica dataBASE
GBI: Glasgow Benefit Inventory
GHABP: Glasgow Hearing Aid Benefit Profile
HINT: Hearing in Noise Test
HUI: Health Utilities Index
ICTRP: International Clinical Trials Registry Platform
IOI-HA: International Outcome Inventory for Hearing Aids
ISRCTN: International Standard Randomised Controlled Trials Number
MEDLINE: Medical Literature Analysis and Retrieval System Online
MEI: Middle ear implant
NCIQ: Nijmegen Cochlear Implant Questionnaire
NIHR: National Institute for Health Research
OlSa: Oldenburg Sentence Test
PICOS: Population, Intervention, Comparator / Control, Outcomes, Setting
PRISMA: Preferred Reporting Items for Systematic Reviews and Meta-Analyses
PROMs: Patient-reported outcome measures
PROSPERO: International Prospective Register of Systematic Reviews
PTA: Pure-tone audiometry
QuickSIN: Quick Speech-in-Noise
SF-36: Medical Outcome Study Short Form 36
SHQ: Spatial Hearing Questionnaire
SPIN: Speech Intelligibility In Noise
SRT: Speech Reception Thresholds
SSD: Single-sided deafness
SSQ: Speech, Spatial and Qualities Scale
THI: Tinnitus Handicap Inventory
TQ: Tinnitus Questionnaire
USA: United Sates of America
VAS: Visual analogue scale
WHO: World Health Organization
WHOQOL-BREF: World Health Organization Quality of Life Short Form Survey
