# Supplementary material for: Systematic review of outcome domains and instruments used in designs of clinical trials for interventions that seek to restore bilateral and binaural hearing in adults with unilateral severe to profound sensorineural hearing loss (‘single-sided deafness’)
Source: Trials. 2021 Mar 20;22:220. doi: 10.1186/s13063-021-05160-5 (PMC7981927; doi:10.1186/s13063-021-05160-5)
Supplement: Supplementary file 2 — Additional file 2. Search syntax – MEDLINE and EMBASE. Search syntax for Excerpta Medica dataBASE (EMBASE) and Medical Literature Analysis and Retrieval System Online (MEDLINE) via OvidSP. [file 13063_2021_5160_MOESM2_ESM.pdf]

Towards a Consensus on Outcome Measures for Interventions that Seek to Restore Bilateral and Binaural Hearing in Adults with Unilateral Severe-to-Profound Hearing Loss: The CROSSSD (Core Rehabilitation Outcome Set for Single Sided Deafness) Study

Search Syntax for Excerpta Medica dataBASE (EMBASE) and Medical Literature Analysis and Retrieval System Online (MEDLINE) via OvidSP:

	Term Type	Search Term	Field Restrictions
**Condition & Causes:**			
1.	MeSH	unilateral hearing loss	
2.	MeSH	acoustic neuroma	
3.	MeSH	sudden hearing loss	
4.	MeSH	meniere disease	
5.	Text	unilateral adj3 hearing loss	title, abstract
6.	Text	unilateral adj3 deafness	title, abstract
7.	Text	single sided adj3 deafness	title, abstract
8.	Text	asymmetric adj3 hearing	title, abstract
9.	Boolean	1 OR 2 OR 3 OR 4 OR 5 OR 6 OR 7 OR 8	
**Interventions:**			
10.	MeSH	bone conduction hearing aid	
11.	MeSH	bone conduction	
12.	MeSH	middle ear implant	
13.	MeSH	cochlear implant	
14.	Text	bone anchored adj2 implant*	title, abstract
15.	Text	bone anchored adj2 aid*	title, abstract
16.	Text	bone conduction adj2 device*	title, abstract
17.	Text	BAHA*	title, abstract
18.	Text	BCD*	title, abstract
19.	Text	contralateral routing adj2 sound*	title, abstract
20.	Text	contralateral routing adj2 signal*	title, abstract
21.	Text	contralateral rerouting adj2 sound*	title, abstract
22.	Text	contralateral rerouting adj2 signal*	title, abstract
23.	Text	contralateral re-routing adj2 sound*	title, abstract
24.	Text	contralateral re-routing adj2 signal*	title, abstract
25.	Text	CROS	title, abstract
26.	Text	BiCROS	title, abstract
27.	Text	middle ear implant*	title, abstract
28.	Text	MEI	title, abstract
29.	Text	auditory implant*	title, abstract
30.	Text	cochlear implant*	title, abstract
31.	Text	transcranial	title, abstract

32.	Text	percutaneous adj2 device	title, abstract
33.	Text	percutaneous adj2 implant	title, abstract
34.	Text	subcutaneous adj2 device	title, abstract
35.	Text	subcutaneous adj2 implant	title, abstract
36.	Boolean	10 OR 11 OR 12 OR 13 OR 14 OR 15 OR 16 OR 17 OR 18 OR 19 OR 20 OR 21 OR 22 OR 23 OR 24 OR 25 OR 26 OR 27 OR 28 OR 29 OR 30 OR 31 OR 32 OR 33 OR 34 OR 35	
**Composition:**			
37.	Boolean	9 AND 36	

Commands specific to OvidSP interface:
* = truncated match
MeSH = Medical Subject Headings
Boolean = Operators used to retrieve search terms
adj2 = Adjacency / Proximity, words have to appear within 2 words of each other
