# Supplementary material for: Systematic review of outcome domains and instruments used in designs of clinical trials for interventions that seek to restore bilateral and binaural hearing in adults with unilateral severe to profound sensorineural hearing loss (‘single-sided deafness’)
Source: Trials. 2021 Mar 20;22:220. doi: 10.1186/s13063-021-05160-5 (PMC7981927; doi:10.1186/s13063-021-05160-5)
Supplement: Supplementary file 3 — Additional file 3. Search syntax - CENTRAL. Search syntax for Cochrane Central Register of Controlled Trials (CENTRAL). [file 13063_2021_5160_MOESM3_ESM.pdf]

Towards a Consensus on Outcome Measures for Interventions that Seek to Restore Bilateral and Binaural Hearing in Adults with Unilateral Severe-to-Profound Hearing Loss: The CROSSSD (Core Rehabilitation Outcome Set for Single Sided Deafness) Study

Search Syntax for Cochrane Central Register of Controlled Trials (CENTRAL):

Keyword Search: (PICO) – Title, Abstract, Keywords search
#1 single sided deafness:ti,ab,kw (Word variations have been searched)
#2 unilateral hearing loss:ti,ab,kw (Word variations have been searched)
#3 unilateral deafness:ti,ab,kw (Word variations have been searched)
#4 asymmetric hearing:ti,ab,kw (Word variations have been searched)
#5 'acoustic neuroma':ti,ab,kw (Word variations have been searched)
#6 'sudden hearing loss':ti,ab,kw (Word variations have been searched)
#7 meniere disease:ti,ab,kw (Word variations have been searched)
#8 (unilateral next/3 hearing loss):ti,ab,kw (Word variations have been searched)
#9 (unilateral next/3 deafness):ti,ab,kw (Word variations have been searched)
#10 (single sided next/3 deafness):ti,ab,kw (Word variations have been searched)
#11 (asymmetric next/3 hearing) .:ti,ab,kw (Word variations have been searched)

MeSH Terms: (PICO)
#12 MeSH descriptor: [Hearing Loss, Unilateral] explode all trees
#13 MeSH descriptor: [Neuroma, Acoustic] explode all trees
#14 MeSH descriptor: [Hearing Loss, Sudden] explode all trees
#15 MeSH descriptor: [Meniere Disease] explode all trees

(PICO) Combinations
#16: #1 OR #2 OR #3 OR #4 OR #5 OR #6 OR #7 OR #8 OR #9 OR #10 OR #11 OR #12 OR #13 OR #14 OR #15

Keyword Search: (PICO) – Title, Abstract, Keywords search
#17 Bone Anchored Hearing Aid:ti,ab,kw (Word variations have been searched)
#18 Middle Ear Implant:ti,ab,kw (Word variations have been searched)
#19 Contralateral Routing:ti,ab,kw (Word variations have been searched)
#20 Cochlear Implant:ti,ab,kw (Word variations have been searched)
#21 bone conduction device:ti,ab,kw (Word variations have been searched)

MeSH Terms: (PICO)
#22 MeSH descriptor: [Hearing Aids] explode all trees
#23 MeSH descriptor: [Bone Conduction] explode all trees
#24 MeSH descriptor: [Ossicular Prosthesis] explode all trees
#25 MeSH descriptor: [Cochlear Implants] explode all trees
#26 MeSH descriptor: [Cochlear Implantation] explode all trees

(PICO) Combinations
#27: #17 OR #18 OR #19 OR #20 OR #21 OR #22 OR #23 OR #24 OR #25 OR #26

Both Searches P and I (PICO) combined for final yield.
