# Supplementary material for: Systematic review of outcome domains and instruments used in designs of clinical trials for interventions that seek to restore bilateral and binaural hearing in adults with unilateral severe to profound sensorineural hearing loss (‘single-sided deafness’)
Source: Trials. 2021 Mar 20;22:220. doi: 10.1186/s13063-021-05160-5 (PMC7981927; doi:10.1186/s13063-021-05160-5)
Supplement: Supplementary file 4 — Additional file 4. Title and abstract screening. Methodology for CROSSSD title and abstract article screening. [file 13063_2021_5160_MOESM4_ESM.pdf]

Methodology for CROSSSD Title and Abstract article screening

CROSSSD Study:
Core Rehabilitation Outcome Set for Single Sided Deafness Study

Aim:
Towards a Consensus on Outcome Measures for Interventions that Seek to Restore Bilateral and Binaural Hearing in Adults with Unilateral Severe-to-Profound Hearing Loss

PROSPERO:
https://www.crd.york.ac.uk/PROSPERO/display_record.php?RecordID=84274

PICO Criteria:
A detailed description of PICO criteria can be found in the table below.

A numbering system has been adopted and it is used to screen articles on the basis of Title and Abstract only in initial screening phase. Please give each article only one code.

1. **Relevant- Include**
   It definitely fits all the CROSSSD PICO criteria.
   Even if the outcomes measured e.g. speech testing / questionnaires are not explicitly mentioned or clearly listed in the title / abstract please code as n = 1; i.e. do not exclude if unsure of outcomes employed specifically.

2. **Unsure / Possibly relevant**
   Not entirely clear if it definitely fits all the CROSSSD PICO criteria.
   Most commonly the title and abstract describe 'implanted participants' but do not explicitly state they have bilateral or unilateral deafness.
   Even if it seems like they probably have bilateral deafness, please code as n = 2 so that we can be confident at ruling out at the full-text screening stage.

3. **Irrelevant- Out of scope**
   Does not fit the CROSSSD PICO criteria e.g. middle ear surgery is used as an intervention.
   If this reason is given by one Screener, but a more specific reason is given by the other Screener, then go with the more specific reason.
   If a study is an animal study exclude using code n = 3.
   If a study is purely a cost utility / effectiveness study exclude using code n = 3.

4. **Irrelevant- P, not SSD, not SNHL**
   Participants do not fit the SSD definition e.g. have mixed loss, or moderate severity.
   If the title / abstract are not explicit about whether patients have SSD or bilateral deafness, then pass the article to full text screening by coding n = 2.
   If the study is referring to NF2 (Neurofibromatosis Type II) patients; these are known to have either unilateral and/or bilateral deafness, so if not clear of the participants audiometric characteristics in title / abstract please code n = 2 so the full text can be retrieved to clarify.
   'Aural atresia' describes a conductive or mixed hearing loss, not SNHL, thus can also be excluded with code n = 4.

5. **Irrelevant- P, not adults**
   All participants groups who are under the age of 18 years.
   If the title / abstract indicate that both children and adults are included, then code the article as n = 1 to pass to full text screening.

6. **Irrelevant- Intervention**
   Intervention is not any technological intervention designed to restore bilateral (two-sided) or binaural (both ears) hearing in order to address the impact of SSD in adults.

Auditory Brainstem Implants and the Soundbite studies should be coded as n = 1 if they fit the rest of the PICO criteria.

7. **Irrelevant- Design type**
   Study cannot be included because it is e.g. a case study or a literature review.
   Retrospective case series-exclude by coding n = 7.
   Systematic reviews should be coded as n = 1 if they fit the rest of the PICO criteria.

8. **Incomplete reference**
   The Title and/or Abstract were not pulled through to EndNote and cannot be found online.

9. **Abstract not accessible**
   Title is available but no abstract is available e.g. it's a book chapter, it's a correspondence with no abstract or from a dated publication that did not include abstracts.

Coding Strategy:
Please number according to the first applicable reason identified (moving hierarchically from code 1 to code 9 in that order).

e.g. *Conductive hearing loss and cranial osseous dysplasia secondary to neurofibromatosis type 1: A case report and literature review.* Should be coded n = 4 because the population is not SNHL (not n = 7, as per Design Type being a Case Review).

The only exception to this has been for some articles that could potentially be coded as n = 3 (out of scope).
-If the article is completely out of scope, then code as n = 3.
-If a more specific code can be given e.g. n = 4,5,6; then use the lowest number on the list (n = 4), hierarchically.
i.e. The most specific code and the one earlier on the hierarchy of codes should be used.

PICO for INCLUSIONS in detail:

**P Inclusions**	Adult male and female participants with **SSD**, of minimum age 18 years. Participants with a diagnosis of congenital or acquired **SNHL** of **threshold severity worse than 70dB HL** at audiometric frequencies ranging from **1-4 kHz** on the worse-hearing ear and normal hearing thresholds on the better hearing ear, defined as pure tone average of ≤30dB HL.
**I Inclusions**	**Bilateral (Re-Routing Interventions)**: (1) Contralateral Routing of Signals (CROS) hearing aid devices and (2) Bone Anchored Hearing Aids (BAHA). **Binaural (Direct Stimulation of Impaired Ear):** (1) Middle Ear Implants (MEI) and (2) Cochlear Implants (CI).<br>We also include studies that evaluate accessories for the above devices e.g. Roger Pen, controlled studies that compare for example different types of cochlear implants, studies comparing different algorithms or fitting strategies or insertion depth. Auditory Brainstem Implants and the Soundbite should also be included.
**C Inclusions**	Normal hearing group only.
**Design Type Inclusion**	Randomised Controlled Trials, Quasi-Randomised Controlled Trials, Before & After Studies, Non-Randomised Controlled Trials, Cross-Over Studies. Trial registrations of such ongoing studies. Systematic Reviews.

PICO for EXCLUSIONS in detail:

**P Exclusions**	Participants younger than 18 years of age. Participants with a diagnosis of mild-moderate asymmetrical hearing losses who are candidates for hearing aid amplification in the ‘normal’ / ‘near-normal’ / ‘better’ hearing ear. Participants with a diagnosis of mild-moderate conductive or mixed hearing loss.
**I Exclusions**	Conventional hearing aids.
**C Exclusions**	Normal hearing group only.
**Design Type Exclusion**	Case control studies, Cohort studies, Literature reviews, Practice Guidelines, Expert Opinions, Case series, Case reports, Book chapters, Conference papers, Manufacturers’ articles, Animal studies. Also excludes studies that use predictive modelling (prognostic factors). Also excludes Editorials and Letters to the Editor. Also excludes retrospective studies of clinical cases.
