# Supplementary material for: Systematic review of outcome domains and instruments used in designs of clinical trials for interventions that seek to restore bilateral and binaural hearing in adults with unilateral severe to profound sensorineural hearing loss (‘single-sided deafness’)
Source: Trials. 2021 Mar 20;22:220. doi: 10.1186/s13063-021-05160-5 (PMC7981927; doi:10.1186/s13063-021-05160-5)
Supplement: Supplementary file 5 — Additional file 5. Data extraction. CROSSSD data extraction protocol. [file 13063_2021_5160_MOESM5_ESM.pdf]

CROSSSD data extraction protocol

CROSSSD Study:
Core Rehabilitation Outcome Set for Single Sided Deafness Study

Aim:
Towards a Consensus on Outcome Measures for Interventions that Seek to Restore Bilateral and Binaural Hearing in Adults with Unilateral Severe-to-Profound Hearing Loss

PROSPERO:
https://www.crd.york.ac.uk/PROSPERO/display_record.php?RecordID=84274

Scope:
This guidance document was developed to ensure consistency of data extraction procedures across reviewers.

Methodology:
Data collection will be conducted electronically on an Excel spreadsheet and will be initially piloted independently by RK and DAH with five studies. The two reviewers will then meet to discuss the suitability of the selected data fields. If indicated, the fields will be amended accordingly or additional fields will be included to capture additional information that is deemed important. A further five studies will then be piloted using the amended spreadsheet and if both reviewers deem the information captured adequate the 'finalised' version of the Data Extraction Spreadsheet will be developed. If not another five studies will be piloted until RK and DAH are happy with all the information captured.

Full data collection for all articles (n=70) that fit the PROSPERO described criteria will be conducted independently and in duplicate by RK, DAH and/or PTK and data will subsequently be compared and compiled into a single data extraction record. In cases of disagreement on data extracted, an arbitrator (DAH or PTK) will be asked to take a decision with regards to data extraction.

General framework:
The following four basic categories of data will be extracted:

a. **Methodological and substantive features:** Including source of the study, year of publication, type of research design etc. Documenting these features should help to relate these characteristics to the study findings (Brown *et al.,* 2003) and will help to explore relationships in the data (Popay *et al.,* 2006).
b. **Study quality:** Including assessment of the robustness of the study, such as the use of validated instruments (Kitterick *et al.,* 2016) utilised to evaluate the effects of interventions; and if the reported outcomes were on the basis of the instruments' measurement properties (Mokkink, *et al.,* 2010).
c. **Intervention descriptors:** Including the two types of hearing interventions (bilateral and binaural) as described by the CROSSSD study aims, and relevant clinical issues, such as cause of SSD, patients' age range, time between SSD diagnosis and implementation of intervention etc (Brown *et al.,* 2003).
d. **Outcome measures:** Based on PROSPERO, all outcomes relating to the interventions of interest will be considered; such as primary and secondary outcomes defined by study researchers, harms etc.

Specific Data Fields:
Selected data items were decided on the basis of the PICOS criteria as outlined in PROSPERO and guided by Popay *et al.,* (2006) principles and Hall *et al.,* (2016) methodology. The selected fields also relate to trial design and methodology adopted to measure the effects of a particular SSD intervention. The key headings (Table 1) for data extraction were developed using thematic analysis and with a scoping review of the current literature e.g. Van Zon *et al.,* (2015), Kitterick *et al.,* (2016), Van de Heyning *et al.,* (2016).

Table 1: Excel Spreadsheet dataset column headings and their descriptions

Col	Dataset column heading	Description
**A**	Study ID	As allocated by the original CROSSSD screening spreadsheet
**B**	Authors	As listed on the published article. For registered trials give the corresponding

		author
**C**	Year of publication	As listed on the published article. For registered trials give the date they were published online. If ePub give the date they were published online
**D**	Journal	Give the journal name the study was published in or the Name of the Registry for registered trials
**E**	Study Title	Full title of the article or registered trial
**F**	Corresponding author	As listed on the article or the principal investigator for registered clinical trials if a corresponding author is not listed
**G**	Email of Corresponding Author	As documented on the publication, or if not available endeavor to find online via the author's institution website
**H**	Country/ies Study Conducted	Write the country name only. If a multi-centre study indicate by writing 'multi-centre'. If the study is conducted at multiple countries list all countries e.g. *If in Lille / Lyon / Paris / Rennes / Tours, France write 'France, multi-centre' or if Lille / Rennes, France & Antwerp / Yvoir, Belgium write 'France & Belgium multi-centre'*
**I**	Study Start Date	Write in format MM/ YYYY e.g. *January 2009 is 01/2009*. If the month is not available write in format 00/YYYY
**J**	Study Design / Type of Study	Choose one of: Randomised Controlled Trials, Quasi-Randomised Controlled Trials, Before & After Study, Non-Randomised Controlled Trials, Cross-Over Studies, Clinical Trial Registration, Systematic Review. Any relevant / additional details should be noted in Column AJ (Notes Column)
**K**	SSD Cause / Participant Characteristics	Choose one of: Congenital, Acoustic Neuroma, Ménière's disease, Sudden Hearing Loss, Trauma, Unknown. If participants included have SSD due to different causes pls list all of the causes with number of participants noted e.g. *acoustic neuroma x5, Trauma x6* etc. If cause is not stated please document as 'Not stated'
**L**	Number of Participants	List the number of enrolled participants, if several groups, including controls; please write the number of participants in each group e.g. *Grp 1 trialing CROS in 10 patients and Grp 2 trialing a Cochlear Implant in 5 patients write CROS n=10 and CI n=5*
**M**	Participant Age Range	Record in years, if not stated please document as 'Not stated'. If several groups included please record for the SSD / UHL group only
**N**	Participant Mean Age	Record in years, if not stated please document as 'Not stated'
**O**	Age Standard Deviation	If able to calculate using participant characteristics please do so. If not calculated and unable to calculate please document as 'Not stated'. If several groups included please record for the SSD / UHL group only
**P**	Time since SSD Diagnosis	Record when the participants were diagnosed with SSD e.g. *5 years prior to recruitment in the study.* If several groups included please record for the SSD / UHL group only
**Q**	Study Primary Objective	Please copy exactly as defined by authors
**R**	Study Secondary Objective	Please copy exactly as defined by authors
**S**	Type of Intervention	Choose one of: Contralateral Routing of Signals (CROS) hearing aid devices, Bone Anchored Hearing Aids (BAHA), Middle Ear Implants (MEI), Cochlear Implants (CI), Auditory Brainstem Implant (ABI), Soundbite or Adhere. If specific details are given about the intervention e.g. for BAHA if Percutaneous or Subcutaneous (Attract) devices were used please specify which one in the Notes Section (Column AJ), if both were used please list both. For MEI if BoneBridge or SoundBridge was used please specify which one, if both were used please list both. If a Grp 1 was given a CI programmed with X algorithm and was compared to Grp 2 that was given CIs with Y algorithm please record as 'CI + X algorithm' in the Notes section (Column AJ). If there was several groups of participants, given different interventions please list all interventions used
**T**	Intervention	Choose one of: CROS, BAHA, MEI, CI, ABI, Soundbite or Adhere. If different

	Comparator	programming strategies were used please list them e.g. *If a Grp 1 was given a CI programmed with X algorithm and was compared to Grp 2 that was given CIs with Y algorithm please record as 'CI + Y algorithm'*
**U**	Implementation of Intervention	Please record in months or years, on average (mean value); how long after the onset of SSD the intervention was implemented. e.g. if a participant was diagnosed with SSD in 2002 and was enrolled in an interventions study and given an intervention in 2006, then record 4 years. If not stated please document as 'Not stated'
**V**	Primary Outcome Domain: The 'WHAT'	Please copy exactly as described by authors e.g. *Localisation*. If the authors do not explicitly describe the outcome domain as 'Primary' but it is assumed to be a primary outcome please indicate with a '?' e.g. *Localisation?*
**W**	Primary Outcome Measure: The 'HOW'	Record the Outcome Measure e.g. *AB Wordlists in Quiet*. If more than one measure was used e.g. *AB Wordlists in Quiet and QuickSIN testing*, please insert extra rows and record the data in different cells. Please keep the description of the instruments used succinct e.g. *Localisation with use of a 33-loudspeaker array at the horizontal plane*; no need for a full description e.g. *The ability to localize sounds in the horizontal plane was measured in a conventional room (not sound treated) typical of an office. For sound localization testing, all loudspeakers (1, 2, 3, 4, 5, 6) faced towards the participant. Thirty six responses were obtained for each participant.* If questionnaire subsections were measured separately as opposed to the global score please insert extra rows and record as separate Outcome Measures e.g. *If the SSQ questionnaire was used and they measured the Speech, Spatial and Qualities Domain record as 3 different measures i.e. Speech domain subscale of the SSQ, Spatial domain subscale of the SSQ, Qualities domain subscale of the SSQ.*
**X**	Primary Outcomes Measurement Time Frame	Record how long the participants had the intervention for before they were 'tested'. e.g. if participants were implanted with a CI and were enrolled in a study to measure CI outcomes 3 months post implantation please document as '*3 mths*'. If all measurements were conducted on the same day, record as 'Single session'. If several measurements were taken e.g. at 3 months, 6 months, 9 months please list all time frames i.e. *3, 6, 9 mths*. If exact time-frame was not stated please document as 'Not Stated'
**Y**	Secondary Outcome Domain: The 'WHAT'	As per Primary Outcome domain instructions (Column V), but for secondary
**Z**	Secondary Outcome Measure: The 'HOW'	As per Primary Outcome measure instructions (Column W), but for secondary
**AA**	Secondary Outcome Measurement Time Frame	As per Primary Outcomes Measurement Time Frame (Column X), but for secondary
**AB**	Is this a prospective trial registration or published protocol?	Choose Yes (Y) or No (N). Prospective Trial Registration is defined as e.g. https://clinicaltrials.gov/ct2/show/record/NCT02105441, a record in a Clinical Trails Registry. Published Protocol is defined as e.g. https://trialsjournal.biomedcentral.com/articles/10.1186/1745-6215-14-70, a publication in a peer reviewed journal
**AC**	Is this a report of study findings?	Choose Yes (Y) or No (N). Choose N if e.g. the record is a protocol of the intendent study
**AD**	Where there is multiple records for the same study, has the data extraction process been linked?	Choose Yes (Y) or No (N) or Not applicable (N/A). Chose Y if e.g. both the study protocol in the clinical trials registry and the peered reviewed published record were yielded
**AE**	Was quality assessed in terms of the consistency with which outcomes are	Choose Yes (Y) or No (N). Comment on details e.g. APHAB questionnaire was used to measure Listening Difficulty vs QoL

	reported and described within a manuscript?	
**AF**	Were outcomes reported prospectively through trial registration or a published protocol?	Choose Yes (Y) or No (N) or Not applicable (N/A). Choose Y if e.g. The research question and inclusion criteria were established and published before the study was conducted. Choose N/A if e.g. the record you are extracting data from is a publication of the protocol
**AG**	Are the outcomes reported consistently between protocol / registration and study report?	If applicable, comment on e.g. *Clinical Trial registration stated 80 target sample size, but only 56 recruited. Clinical Trial Registration states additional primary outcome is Tinnitus questionnaire (not defined, at 4 and 8 weeks) and additional secondary outcomes are 1- complication including headache measured using check list (not defined) at 4 weeks, 2- complication including vertigo measured using check list (not defined) at 4 weeks*
**AH**	Are there any conflicts of interest noted by the authors?	Choose Yes (Y), No (N) or Not stated, and please give details e.g. If declared that there were no conflicts of interest, record as N. If funding was provided by the hearing company involved e.g. *Funding provided by Sonitus Medical, Inc., San Mateo, CA. The authors have no other funding, financial relationships, or conflicts of interest to disclose*; please choose Y and copy the details as described by the authors
**AI**	Notes on Study Design	Any other study design features that were different e.g. *Recruited 9 but only analysed data for 8 participants*
**AJ**	Notes	Please note anything you feel is relevant to consider e.g. If a clinical trial registry did not recruit any participants or if you have any queries

Notes:
> If unable to find the data for columns as defined, please document as 'Cannot find'
> Generally, if data is not documented in the publication please record as 'Not Stated'

Systematic Reviews: The yielded systematic review records will be reviewed for the individual articles included. All included articles of each systematic review will be assessed independently for eligibility as per set CROSSSD study PICOS criteria. They will then be double-coded according to the original CROSSSD criteria. If any were missed by the original search the Titles / Abstracts will be coded and if necessary the full text PDFs will be retrieved and coded accordingly by two independent reviewers. Data will be extracted from all new (if any) identified records.

Quality Assessment:
The validity of the conclusions of a systematic review depend on the quality of the included primary studies (Downs & Black, 1998). Assessing the quality of studies is defined as an assessment of 'the likelihood of the trial design to generate unbiased results that are sufficiently precise and allow application in clinical practice' (Verhagen *et al.*, 2001). In their systematic review of management options for unilateral hearing loss in children, Appachi *et al.,* (2017) successfully utilised the validated Newcastle-Ottawa Scale (NOS) to assess the quality of the studies reviewed. When NOS is utilised, the reviewer is instructed to assign points when quality domains are present, thus permitting the calculation of overall 'quality scores'. However Oremus *et al.,* (2012) report 'poor to fair' inter-rater reliability only 'fair to excellent' test-rested reliability especially when inexperienced raters are scoring the studies. Furthermore, Higgins *et al.,* (2011) state that quality scales and scale scores are not appropriate tools to utilise when assessing study quality. It considered that the Cochrane Collaboration tool for assessing risk of bias is utilised, with the following types of bias recorded (Higgins *et al.,* 2017):

Table 8.4.a: A common classification scheme for bias

Type of bias	Description	Relevant domains in the Cochrane 'Risk of bias' tool
Selection bias	Systematic differences between baseline characteristics of the groups that are compared	• Sequence generation<br>• Allocation concealment
Performance bias	Systematic differences between groups in the care that is provided, or in exposure to factors other than the interventions of interest	• Blinding of participants and personnel<br>• Other potential threats to validity
Detection bias	Systematic differences between groups in how outcomes are determined	• Blinding of outcome assessment<br>• Other potential threats to validity
Attrition bias	Systematic differences between groups in withdrawals from a study	• Incomplete outcome data
Reporting bias	Systematic differences between reported and unreported findings	• Selective outcome reporting (see also Chapter 10)

However, there is a large variability in study designs and types yielded by the search. These included Randomised Controlled Trials for which the Cochrane tool is tailored for; but also Quasi-Randomised Controlled Trials, Before and After Studies, Non-Randomised Controlled Trials, Cross-Over Studies, Clinical Trial Registrations and Systematic Reviews. Therefore, utilizing the Cochrane tool would not effectively address bias in all types of study designs.

Consequently, Risk of bias will be assessed by analysing the reporting of outcomes both within and across manuscripts reporting study findings. Quality will be assessed in terms of the consistency with which outcomes are reported and described within a manuscript (Column AE), whether outcomes were reported prospectively through trial registration or published protocol (Column AF), and whether outcomes are reported consistently between protocol / registration and study report (Column AG).

The quality of a pilot sample of articles (n=5) will be conducted initially to ensure that criteria are applied consistently by two independent reviewers and that consensus can be reached between reviewers. Following this the Data Extraction Protocol will be modified accordingly. A second batch of n=5 studies will be piloted next to ensure the revised protocol is thoroughly covering all aspects. If the reviewers are happy with the consistency and outcomes they will proceed with data extraction of the rest of the records.

The reviewers will consider blinding (to authors, institutions, journals and study results) when conducting the quality assessment. This will depend on the resources available at the time, although there is limited evidence that blinded assessments is significantly beneficial (Kjaergard *et al.,* 2011).

The consolidated record data (e.g. outcome descriptors, published primary / secondary findings) collated from the review will be qualitatively and critically analysed for consistency of outcome reporting by at least two independent reviewers. If consensus cannot be reached on whether outcomes have been reported consistently

then disagreements will be resolved by consensus with a third reviewer. Findings will be reported using a narrative synthesis. The quality of a study will not affect its inclusion in the synthesis.

Sensitivity Analysis:
A subgroup of studies (n=27) had minor differences form the PROSPERO protocol for example in the sample of actual recruited subjects a small number did not meet the explicit threshold criteria for SSD, despite the overall intention to recruit SSD patients. These have been coded as '1a' so that a sensitivity analysis can be conducted to identify whether this subgroup of studies differ in their outcomes measured. If not, then the outcomes information will be pooled across all studies.

Consolidation of Studies:
A single common extraction will be utilized for e.g. Clinical Trial Registrations and/or Protocols which describe the same study. These studies will be identified and clearly marked. Quality assessment will be undertaken and will be compared for these separately.

References:

Appachi, S., Specht, J.L., Raol, N., Lieu, J.E., Cohen, M.S., Dedhia, K. & Anne, S. (2017). Auditory outcomes with hearing rehabilitation in children with unilateral hearing loss: a systematic review. *Otolaryngol Head Neck Surg*, *157*(4), 565-571.

Downs, S. H., & Black, N. (1998). The feasibility of creating a checklist for the assessment of the methodological quality both of randomised and non-randomised studies of health care interventions. *J Epidemiol Community Health*, *52*, 377–384.

Brown, S.A., Upchurch, S.L. & Acton, G.J., (2003). A framework for developing a coding scheme for meta-analysis. *West J Nurs Res*, *25* (2), 205-222.

Hall, D.A., Haider, H., Szczepek, A.J., Lau, P., Rabau, S., Jones-Diette, J., Londero, A., Edvall, N.K., Cederroth, C.R., Mielczarek, M. & Fuller, T. (2016). Systematic review of outcome domains and instruments used in clinical trials of tinnitus treatments in adults. *Trials*, 17(1), 270-289.

Higgins, J.P., Altman, D.G., Gøtzsche, P.C., Jüni, P., Moher, D., Oxman, A.D., Savović, J., Schulz, K.F., Weeks, L. & Sterne, J.A. (2011). The Cochrane Collaboration's tool for assessing risk of bias in randomised trials. *BMJ*, *343*, p.d5928.

Higgins, J. P. T., Altman, D. G., Sterne, J. A. C. (editors). Chapter 8: Assessing risk of bias in included studies. In: Higgins, J. P. T., Churchill, R., Chandler, J., Cumpston, M. S. (editors), Cochrane Handbook for Systematic Reviews of Interventions version 5.2.0 (updated June 2017), Cochrane, 2017. Available: www.training.cochrane.org/handbook.

Kitterick, P. T., Smith, S. N., & Lucas, L. (2016). Hearing instruments for unilateral severe-to-profound sensorineural hearing loss in adults: a systematic review and meta-analysis. *Ear Hear,* 37(5), 495-507.

Kjaergard, L.L., Villumsen, J. and Gluud, C. (2001). Reported methodologic quality and discrepancies between large and small randomized trials in meta-analyses. Ann Intern Med, 135(11), 982-989.

Mokkink, L.B., Terwee, C.B., Patrick, D.L., Alonso, J., Stratford, P.W., Knol, D.L., Bouter, L.M. & De Vet, H.C., (2010). The COSMIN checklist for assessing the methodological quality of studies on measurement properties of health status measurement instruments: an international Delphi study. *Qual Life Res*, *19* (4), 539-549.

Oremus, M., Oremus, C., Hall, G.B., McKinnon, M.C. and ECT & Cognition Systematic Review Team (2012). Inter-rater and test–retest reliability of quality assessments by novice student raters using the Jadad and Newcastle–Ottawa Scales. *BMJ Open*, *2*(4), e001368.

Popay, J., Roberts, H., Sowden, A., Petticrew, M., Arai, L., Rodgers, M., Britten, N., Roen, K. & Duffy, S., (2006). Guidance on the conduct of narrative synthesis in systematic reviews. *A product from the ESRC methods*

programme Version, *1*, p.b 92.

Van de Heyning, P., Távora-Vieira, D, Mertens, G., Van Rompaey, V., Rajan, G. P., Müller, J., Hempel, J. M, Leander, D., Polterauer, D., Marx, M., Usami, S. I., Kitoh, R., Miyagawa, M., Moteki, H., Smilsky, K., Baumgartner, W. D., Keintzel, T. G., Sprinzl, G. M., Wolf-Magele, A., Arndt, S., Wesarg, T., Zirn, S., Baumann, U., Weissgerber, T., Rader, T., Hagen, R., Kurz, A., Rak, K., Stokroos, R., George, E., Polo, R., Medina, M. D. M., Henkin, Y., Hilly, O., Ulanovski, D., Rajeswaran, R., Kameswaran, M., Di Gregorio, M. F. & Zernotti, M. E. (2016). Towards a Unified Testing Framework for Single-Sided Deafness Studies: A Consensus Paper. *Audiol Neurootol.* 21(6): 391-398.

Van Zon, A., Peters, J.P., Stegeman, I., Smit, A.L. and Grolman, W. (2015). Cochlear implantation for patients with single-sided deafness or asymmetrical hearing loss: a systematic review of the evidence. *Otol Neurotol*, 36 (2), 209-219.

Verhagen, A.P., de Vet, H.C., de Bie, R.A., Boers, M. & van den Brandt, P.A. (2001). The art of quality assessment of RCTs included in systematic reviews. *J Clin Epidemiol*, *54*(7), 651-654.

Consulted:

Higgins, J. P. T. & Green S (2011). Cochrane Handbook for Systematic Reviews of Interventions Version 5.1. The Cochrane Collaboration. Available from www.cochrane-handbook.org

Systematic Reviews, a Guide: Data Extraction. Available from: http://researchguides.ebling.library.wisc.edu/systematic-reviews/author/data

Systematic Reviews: Reporting the quality / risk of bias. Available from: http://libguides.gwumc.edu/c.php?g=27797&p=170451

Systematic Reviews & Meta-analyses: Data Extraction. Available from: https://guides.lib.vt.edu/SystematicReviews/DataExtraction
