# Supplementary material for: Systematic review of outcome domains and instruments used in designs of clinical trials for interventions that seek to restore bilateral and binaural hearing in adults with unilateral severe to profound sensorineural hearing loss (‘single-sided deafness’)
Source: Trials. 2021 Mar 20;22:220. doi: 10.1186/s13063-021-05160-5 (PMC7981927; doi:10.1186/s13063-021-05160-5)
Supplement: Supplementary file 6 — Additional file 6. References for all included records. List of records that explicitly fitted the systematic review inclusion criteria as defined by our PROSPERO protocol and were included in the systematic review data extraction (n = 78), those that closely fitted and were included for sensitivity analysis only (n = 31), and the 12 systematic reviews. [file 13063_2021_5160_MOESM6_ESM.pdf]

Additional file 06. List of records (n=78) that explicitly fitted the systematic review inclusion criteria as defined by our PROSPERO protocol, and were included in the systematic review data extraction; and those (n=31) that closely fitted and were included for sensitivity analysis only. The list of systematic reviews (n=12) that were reviewed is also included.

Records (n=78) that fitted all inclusion criteria and were included in the data extraction:

Agterberg, M.J.H., Snik, A.F.M., Van de Goor, R.M.G., Hol, M.K.S., & Van Opstal, A.J. (2019). Sound-localization performance of patients with single-sided deafness is not improved when listening with a bone-conduction device. *Hear Res*. **372**: 62–68.

Ahmed, M.F., & Khater, A. (2017). Tinnitus suppression after cochlear implantation in patients with single-sided deafness. *Egypt J Otolaryngol*. **33**: 61.

Arndt, S., Aschendorff, A., Laszig, R., Beck, R., Schild, C., Kroeger, S., Ihorst, G., & Wesarg, T. (2011). Comparison of pseudobinaural hearing to real binaural hearing rehabilitation after cochlear implantation in patients with unilateral deafness and tinnitus. *Otol Neurotol*. **32**: 39–47.

Arndt, S., Laszig, R., Aschendorff, A., Beck, R., Schild, C., Hassepass, F., Ihorst, G., Kroeger, S., Kirchem, P., & Wesarg, T. (2011). [Unilateral deafness and cochlear implantation: audiological diagnostic evaluation and outcomes]. *HNO*. **59**: 437–446.

Arndt, S., Laszig, R., Aschendorff, A., Hassepass, F., Beck, R., & Wesarg, T. (2017). [Cochlear implant treatment of patients with single-sided deafness or asymmetric hearing loss. German version]. *HNO*. **65**: 586–598.

Bance, M. (2012). A comparison between wireless CROS and bone-anchored hearing devices for single-sided deafness: A pilot study. In: ClinicalTrials.gov. https://clinicaltrials.gov/ct2/show/NCT01715948?cond=NCT01715948&rank=1. Accessed 10 Aug 2020.

Bosman, A.J., Hol, M.K.S., Snik, A.F.M., Mylanus, E.A.M., & Cremers, C.W.R.J. (2003). Bone-anchored Hearing Aids in Unilateral Inner Ear Deafness. *Acta Otolaryngol*. **123**: 258–260.

Carlson, M.L. (2014). Cochlear implantation among adults and older children with unilateral or asymmetric hearing loss. In: ClinicalTrials.gov. https://clinicaltrials.gov/ct2/show/NCT02105441. Accessed 10 Aug 2020.

Choi, J.E., Ma, S.M., Park, H., Cho, Y.S., Hong, S.H., & Moon, J. (2019). A comparison between wireless CROS/ BiCROS and soft-band BAHA for patients with unilateral hearing loss. *PLoS One*. **14**: e0212503.

Desmet, J.B.J., Wouters, K., De Bodt, M., & Van de Heyning, P. (2012). Comparison of 2 implantable bone conduction devices in patients with single-sided deafness using a daily alternating method. *Otol Neurotol*. **33**: 1018–1026.

Doge, J., Baumann, U., Weissgerber, T., & Rader, T. (2017). Single-sided deafness: Impact of cochlear implantation on speech perception in complex noise and on auditory localization accuracy. *Otol Neurotol*. **38**: e563–e569.

Dunn, C. & Burke, D. (2019). Iowa cochlear implant clinical research center study of SSD using Med-El cochlear implants. In: ClinicalTrials.gov. https://clinicaltrials.gov/ct2/show/NCT03929809. Accessed 10 Aug 2020.

Dwyer, N.Y., Firszt, J.B., & Reeder, R.M. (2014). Effects of unilateral input and mode of hearing in the better ear: self-reported performance using the speech, spatial and qualities of hearing scale. *Ear Hear*. **35**: 126–136.

Firszt, J.B., Holden, L.K., Reeder, R.M., Waltzman, S.B., & Arndt, S. (2012). Auditory abilities after cochlear implantation in adults with unilateral deafness: a pilot study. *Otol Neurotol*. **33**: 1339–1346.

Galvin, J.J., Fu, Q.J., Wilkinson, E.P., Mills, D., Hagan, S.C., Lupo, J.E., Padilla, M., & Shannon, R. V. (2019). Benefits of cochlear implantation for single-sided deafness: Data from the House Clinic-University of Southern California-University of California, Los Angeles Clinical Trial. *Ear Hear*. **40**: 766–781.

Gluth, M.B., Eager, K.M., Eikelboom, R.H., & Atlas, M.D. (2010). Long-term benefit perception, complications, and device malfunction rate of bone-anchored hearing aid implantation for profound unilateral sensorineural hearing loss. *Otol Neurotol*. **31**: 1427–1434.

Gnansia, D., & Frachet, B. (2016). Tinnitus treatment with cochlear implant in single sided deafness. In: ClinicalTrials.gov. https://clinicaltrials.gov/ct2/show/NCT02966366. Accessed 10 Aug 2020.

Grantham, D.W., Ashmead, D.H., Haynes, D.S., Hornsby, B.W.Y., Labadie, R.F., & Ricketts, T.A. (2012). Horizontal plane localization in single-sided deaf adults fitted with a bone-anchored hearing aid (Baha). *Ear Hear*. **33**: 595–603.

Grolman, W. (2014). CINGLE-studie: Cochleaire Implantatie bij siNGLE-sided deafness. In: Netherlands Trial Regist. https://www.trialregister.nl/trial/4457. Accessed 10 Aug 2020.

Grossmann, W., Brill, S., Moeltner, A., Mlynski, R., Hagen, R., & Radeloff, A. (2016). Cochlear implantation improves spatial release from masking and restores localization abilities in single-sided deaf patients. *Otol Neurotol*. **37**: 658–664.

Gurgel, R.K., & Shelton, C. (2013). The SoundBite hearing system: patient-assessed safety and benefit study. *Laryngoscope*. **123**: 2807–2812.

Härkönen, K., Kivekas, I., Kotti, V., Sivonen, V., & Vasama, J.-P. (2017). Hybrid cochlear implantation: quality of life, quality of hearing, and working performance compared to patients with conventional unilateral or bilateral cochlear implantation. *Eur Arch Otorhinolaryngol*. **274**: 3599–3604.

Härkönen, K., Kivekas, I., Rautiainen, M., Kotti, V., Sivonen, V., & Vasama, J.-P. (2015). Single-Sided Deafness: The effect of cochlear Implantation on quality of life, quality of hearing, and working performance. *ORL J Otorhinolaryngol Relat Spec*. **77**: 339–345.

CROSSSD

Häußler, S.M., Köpke, V., Knopke, S., Gräbel, S., & Olze, H. (2020). Multifactorial positive influence of cochlear implantation on patients with single-sided deafness. *Laryngoscope*. **130**: 500–506.
Holder, J.T., O'Connell, B., Hedley-Williams, A., & Wanna, G. (2017). Cochlear implantation for single-sided deafness and tinnitus suppression. *Am J Otolaryngol - Head Neck Med Surg*. **38**: 226–229.
Indeyeva, Y.A., Diaz, A., Imbrey, T., Gao, G., & Coelho, D.H. (2015). Tinnitus management with percutaneous osseointegrated auditory implants for unilateral sensorineural hearing loss. *Am J Otolaryngol*. **36**: 810–813.
Jacob, R., Stelzig, Y., Nopp, P., & Schleich, P. (2011). [Audiological results with cochlear implants for single-sided deafness]. *HNO*. **59**: 453–460.
Kitoh, R., Moteki, H., Nishio, S., Shinden, S., Kanzaki, S., Iwasaki, S., Ogawa, K., & Usami, S.I. (2016). The effects of cochlear implantation in Japanese single-sided deafness patients: Five case reports. *Acta Otolaryngol*. **136**: 460–464.
Kleinjung, T. Single-sided deafness and cochlear implants. In: 2012. https://clinicaltrials.gov/ct2/show/NCT01749592. Accessed 10 Aug 2020.
Krempaska, S., Koval, J., Schmid, C., Pfiffner, F., Kurz, A., & Kompis, M. (2014). Influence of directionality and maximal power output on speech understanding with bone anchored hearing implants in single sided deafness. *Eur Arch Oto-Rhino-Laryngology*. **271**: 1395–1400.
Laske, R.D., Röösli, C., Pfiffner, F., Veraguth, D., & Huber, A.M. (2015). Functional results and subjective benefit of a transcutaneous bone conduction device in patients with single-sided deafness. *Otol Neurotol*. **36**: 1151–1156.
Lee, D.J. (2015). Cochlear implantation for treatment of single-sided deafness. In: ClinicalTrials.gov. https://clinicaltrials.gov/ct2/show/record/NCT02532972. Accessed 10 Aug 2020.
Legris, E., Galvin, J., Roux, S., Gomot, M., Aoustin, J.M., Marx, M., He, S., & Bakhos, D. (2018). Cortical reorganization after cochlear implantation for adults with single-sided deafness. *PLoS One*. **13**: e0204402.
Leterme, G., Bernardeschi, D., Bensemman, A., Coudert, C., Portal, J.J., Ferrary, E., Sterkers, O., Vicaut, E., Frachet, B., & Grayeli, A.B. (2015). Contralateral routing of signal hearing aid versus transcutaneous bone conduction in single-sided deafness. *Audiol Neurotol*. **20**: 251–260.
Linstrom, C.J., Silverman, C.A., & Yu, G.-P.P. (2009). Efficacy of the bone-anchored hearing aid for single-sided deafness. *Laryngoscope*. **119**: 713–720.
Litovsky, R.Y., Moua, K., Godar, S., Kan, A., Misurelli, S.M., & Lee, D.J. (2019). Restoration of spatial hearing in adult cochlear implant users with single-sided deafness. *Hear Res*. **372**: 69–79.
Lorens, A., Kruszyńska, M., Obrycka, A., Skarzynski, P.H., Wilson, B., & Skarzynski, H. (2019). Binaural advantages in using a cochlear implant for adults with profound unilateral hearing loss. *Acta Otolaryngol*. **139**: 153–161.
Marx, M. (2014). Cochlear implantation in single sided deafness and asymmetrical hearing loss: a cost-utility study. In: ClinicalTrials.gov. https://clinicaltrials.gov/ct2/show/NCT02204618. Accessed 10 Aug 2020.
Marx, M., Costa, N.N., Lepage, B., Taoui, S., Molinier, L., Deguine, O., & Fraysse, B. (2019). Cochlear implantation as a treatment for single-sided deafness and asymmetric hearing loss: a randomized controlled evaluation of cost-utility. *BMC Ear, Nose Throat Disord*. **19**: 1.
May, B.J., Bowditch, S., Liu, Y., Eisen, M., & Niparko, J.K. (2014). Mitigation of informational masking in individuals with single-sided deafness by integrated bone conduction hearing aids. *Ear Hear*. **35**: 41–48.
Mertens, G., De Bodt, M., & Van de Heyning, P. (2016). Cochlear implantation as a long-term treatment for ipsilateral incapacitating tinnitus in subjects with unilateral hearing loss up to 10 years. *Hear Res*. **331**: 1–6.
Mertens, G., Gilles, A., Bouzegta, R., & Van De Heyning, P. (2018). A prospective randomized crossover study in single sided deafness on the new non-invasive adhesive bone conduction hearing system. *Otol Neurotol*. **39**: 940–949.
Mertens, G., Punte, A.K., De Ridder, D., & Van De Heyning, P. (2013). Tinnitus in a single-sided deaf ear reduces speech reception in the non tinnitus ear. *Otol Neurotol*. **34**: 662–666.
Miller, R., Hujoel, P., Murray, M., & Popelka, G.R. (2011). Safety of an intra-oral hearing device utilizing a split-mouth research design. *J Clin Dent*. **22**: 159–162.
Moore, B.C.J., & Popelka, G.R. (2013). Preliminary comparison of bone-anchored hearing instruments and a dental device as treatments for unilateral hearing loss. *Int J Audiol*. **52**: 678–686.
Moteki, H., Kitoh, R., & Usami, S. ichi. (2020). The availability of an adhesive bone conduction hearing device: a preliminary report of a single-center experience. *Acta Otolaryngol*. **0**: 1–8.
Mueller, J., Behr, R., Knaus, C., Milewski, C., Schoen, F., & Helms, J. (2000). Electrical stimulation of the auditory pathway in deaf patients following acoustic neurinoma surgery and initial results with a new auditory brainstem implant system. *Adv Otorhinolaryngol*. **57**: 229–235.
Murray, M., Miller, R., Hujoel, P., & Popelka, G.R. (2011). Long-term safety and benefit of a new intraoral device for single-sided deafness. *Otol Neurotol*. **32**: 1262–1269.
Newman, C.W., Sandridge, S.A., & Oswald, L.M. (2010). Relationship between expectations and satisfaction for Baha implant system in patients with single-sided deafness. *Semin Hear*. **31**: 15–27.
Newman, C.W., Sandridge, S.A., & Wodzisz, L.M. (2008). Longitudinal benefit from and satisfaction with the Baha system for patients with acquired unilateral sensorineural hearing loss. *Otol Neurotol*. **29**: 1123–1131.

Niparko, J.K., Cox, K.M., & Lustig, L.R. (2003). Comparison of the bone anchored hearing aid implantable hearing device with contralateral routing of offside signal amplification in the rehabilitation of unilateral deafness. *Otol Neurotol.* **24**: 73–78.
Oeding, K., & Valente, M. (2013). The effectiveness of the directional microphone in the Oticon Medical Ponto Pro in participants with unilateral sensorineural hearing loss. *J Am Acad Audiol*. **24**: 701–713.
Oeding, K., Valente, M., & Kerckhoff, J. (2010). Effectiveness of the directional microphone in the Baha® Divino™. *J Am Acad Audiol*. **21**: 546–557.
Pai, I., Kelleher, C., Nunn, T., Pathak, N., Jindal, M., O'Connor, A.F., & Jiang, D. (2012). Outcome of bone-anchored hearing aids for single-sided deafness: a prospective study. *Acta Otolaryngol*. **132**: 751–755.
Pelusso, C. (2019). Adhear Bone Conduction System. In: ClinicalTrials.gov. https://clinicaltrials.gov/ct2/show/study/NCT03533686. Accessed 10 Aug 2020.
Peter, N., Kleinjung, T., Probst, R., Hemsley, C., Veraguth, D., Huber, A., Caversaccio, M., Kompis, M., Mantokoudis, G., Senn, P., & Wimmer, W. (2019). Cochlear implants in single-sided deafness - clinical results of a Swiss multicentre study. *Swiss Med Wkly*. **149**: w20171.
Pfiffner, F., Kompis, M., Flynn, M., Asnes, K., Arnold, A., & Stieger, C. (2011). Benefits of low-frequency attenuation of Baha(R) in single-sided sensorineural deafness. *Ear Hear*. **32**: 40–45.
Poncet-Wallet, C., Mamelle, E., Godey, B., Truy, E., Guevara, N., Ardoint, M., Gnansia, D., Hoen, M., Saaï, S., Mosnier, I., Lescanne, E., Bakhos, D., & Vincent, C. (2020). Prospective multicentric follow-up study of cochlear implantation in adults with single-sided deafness: Tinnitus and audiological outcomes. *Otol Neurotol*. **41**: 458–466.
Popelka, G.R. (2010). SoundBite hearing system by Sonitus Medical: A new approach to single-sided deafness. *Semin Hear*. **31**: 393–409.
Popelka, G.R., Derebery, J., Blevins, N.H., Murray, M., Moore, B.C.J., Sweetow, R.W., Wu, B., & Katsis, M. (2010). Preliminary evaluation of a novel bone-conduction device for single-sided deafness. *Otol Neurotol*. **31**: 492–497.
Punte, A.K., De Ridder, D., & Van de Heyning, P. (2013). On the necessity of full length electrical cochlear stimulation to suppress severe tinnitus in single-sided deafness. *Hear Res*. **295**: 24–29.
Punte, A.K., Vermeire, K., Hofkens, A., De Bodt, M., De Ridder, D., & Van de Heyning, P. (2011). Cochlear implantation as a durable tinnitus treatment in single-sided deafness. *Cochlear Implants Int*. **12 Suppl 1**: S26-9.
Ryu, N.-G., Moon, I.J., Byun, H., Jin, S.H., Park, H., Jang, K.-S., & Cho, Y.-S. (2015). Clinical effectiveness of wireless CROS (contralateral routing of offside signals) hearing aids. *Eur Arch Otorhinolaryngol*. **272**: 2213–2219.
Saliba, I., Nader, M.-E., El Fata, F., & Leroux, T. (2011). Bone anchored hearing aid in single sided deafness: outcome in right-handed patients. *Auris Nasus Larynx*. **38**: 570–576.
Schafer, Erin C.; Baldus, Nicole; D'Souza, Miranda; Algier, Katherine; Whiteley, Paige; Hill, M. (2013). Behavioral and subjective performance with digital CROS/BiCROS hearing instruments. *J Acad Rehabil Audiol*. **46**: 62–93.
Schmerber, S., Deguine, O., Marx, M., van de Heyning, P., Sterkers, O., Mosnier, I., Garin, P., Godey, B., Vincent, C., Venail, F., Mondain, M., Deveze, A., Lavieille, J.P., & Karkas, A. (2017). Safety and effectiveness of the Bonebridge transcutaneous active direct-drive bone-conduction hearing implant at 1-year device use. *Eur Arch Otorhinolaryngol*. **274**: 1835–1851.
Shannon, R. V. (2014). Cochlear implants for adults with single-sided deafness. In: ClinicalTrials.gov. https://clinicaltrials.gov/ct2/show/NCT02259192. Accessed 10 Aug 2020.
Snapp, H.A., Hoffer, M.E., Liu, X., & Rajguru, S.M. (2017). Effectiveness in rehabilitation of current wireless CROS technology in experienced bone-anchored implant users. *Otol Neurotol*. **38**: 1397–1404.
Snapp, H.A., Holt, F.D., Liu, X., & Rajguru, S.M. (2017). Comparison of speech-in-noise and localization benefits in unilateral hearing loss subjects using contralateral routing of signal hearing aids or bone-anchored implants. *Otol Neurotol*. **38**: 11–18.
Song, J.-J., Kim, K., Sunwoo, W., Mertens, G., de Heyning, P. Van, Ridder, D. De, Vanneste, S., Lee, S.-Y., Park, K.-J., Choi, H., & Choi, J.-W. (2018). Corrigendum: A quantitative electroencephalography study on cochlear implant-induced cortical changes in single-sided deafness with tinnitus. *Front Hum Neurosci*. **12**: 46.
Song, J.-J., Punte, A.K., De Ridder, D., Vanneste, S., & Van de Heyning, P. (2013). Neural substrates predicting improvement of tinnitus after cochlear implantation in patients with single-sided deafness. *Hear Res*. **299**: 1–9.
Syms, Charles & Galow, L. (2013). Evaluation of benefit for treatment of Single Sided Deafness (SSD) between two bone conduction prosthetic devices; osseointegrated implant versus maxilla anchored removable oral appliance ("SoundBite"). In: ClinicalTrials.gov. https://clinicaltrials.gov/ct2/show/NCT01933386. Accessed 10 Aug 2020.
Távora-Vieira, D., Boisvert, I., McMahon, C.M., Maric, V., & Rajan, G.P. (2013). Successful outcomes of cochlear implantation in long-term unilateral deafness: brain plasticity? *Neuroreport*. **24**: 724–729.
Távora-Vieira, D., De Ceulaer, G., Govaerts, P.J., & Rajan, G.P. (2015). Cochlear implantation improves localization ability in patients with unilateral deafness. *Ear Hear*. **36**: e93-8.
Távora-Vieira, D., Marino, R., Acharya, A., & Rajan, G.P. (2015). The impact of cochlear implantation on speech understanding, subjective hearing performance, and tinnitus perception in patients with unilateral severe to profound hearing loss. *Otol Neurotol*. **36**: 430–436.
Wazen, J.J., Ghossaini, S.N., Spitzer, J.B., & Kuller, M. (2005). Localization by unilateral BAHA users. *Otolaryngol - Head Neck Surg*. **132**: 928–932.

Wazen, J.J., Spitzer, J.B., Ghossaini, S.N., Fayad, J.N., Niparko, J.K., Cox, K., Brackmann, D.E., & Soli, S.D. (2003). Transcranial contralateral cochlear stimulation in unilateral deafness. *Otolaryngol Head Neck Surg.* **129**: 248–254.
Weber, B.A., Roush, J., & McElveen, J.T.J. (1992). Application of an implantable bone conduction hearing device to patients with unilateral sensorineural hearing loss. *Laryngoscope*. **102**: 538–542.

Records (n=31) that closely fitted the inclusion criteria and were included for sensitivity analysis only:

Andersen, H.T., Schrøder, S.A., & Bonding, P. (2006). Unilateral deafness after acoustic neuroma surgery: subjective hearing handicap and the effect of the bone-anchored hearing aid. *Otol Neurotol*. **27**: 809–814.
Bernardeschi, D., Russo, F.Y., Nguyen, Y., Vicault, E., Flament, J., Bernou, D., Sterkers, O., & Mosnier, I. (2016). Audiological Results and Quality of Life of Sophono Alpha 2 Transcutaneous Bone-Anchored Implant Users in Single-Sided Deafness. *Audiol Neurootol*. **21**: 158–164.
Buechner, A., Brendel, M., Lesinski-Schiedat, A., Wenzel, G., Frohne-Buechner, C., Jaeger, B., & Lenarz, T. (2010). Cochlear implantation in unilateral deaf subjects associated with ipsilateral tinnitus. *Otol Neurotol*. **31**: 1381–1385.
Dillon, M.T. (2014). Cochlear Implantation in cases of Single-Sided Deafness (CI in SSD). In: ClinicalTrials.gov. https://clinicaltrials.gov/ct2/show/NCT02203305. Accessed 10 Aug 2020.
Dillon, M.T., Buss, E., Anderson, M.L., King, E.R., Deres, E.J., Buchman, C.A., Brown, K.D., & Pillsbury, H.C. (2017). Cochlear implantation in cases of unilateral hearing loss: Initial localization abilities. *Ear Hear*. **38**: 611–619.
Dillon, M.T., Buss, E., Rooth, M.A., King, E.R., Deres, E.J., Buchman, C.A., Pillsbury, H.C., & Brown, K.D. (2017). Effect of cochlear implantation on quality of life in adults with unilateral hearing loss. *Audiol Neurotol*. **22**: 259–271.
Doobe, G., Ernst, A., Ramalingam, R., Mittmann, P., & Todt, I. (2015). Simultaneous labyrinthectomy and cochlear implantation for patients with single-sided Ménière's Disease and profound sensorineural hearing loss. Biomed Res Int. https://doi.org/10.1155/2015/457318.
Hol, M.K.S., Bosman, A.J., Snik, A.F.M., Mylanus, E.A.M., & Cremers, C.W.R.J. (2004). Bone-anchored hearing aid in unilateral inner ear deafness: a study of 20 patients. *Audiol Neurootol*. **9**: 274–281.
Hol, M.K.S., Bosman, A.J., Snik, A.F.M., Mylanus, E.A.M., & Cremers, C.W.R.J. (2005). Bone-anchored hearing aids in unilateral inner ear deafness: an evaluation of audiometric and patient outcome measurements. *Otol Neurotol*. **26**: 999–1006.
Hol, M.K.S., Kunst, S.J.W., Snik, A.F.M., & Cremers, C.W.R.J. (2010). Pilot study on the effectiveness of the conventional CROS, the transcranial CROS and the BAHA transcranial CROS in adults with unilateral inner ear deafness. *Eur Arch Otorhinolaryngol.* **267**: 889–896.
Hol, M.K.S., Kunst, S.J.W., Snik, A.F.M., Bosman, A.J., Mylanus, E.A.M., & Cremers, C.W.R.J. (2010). Bone-anchored hearing aids in patients with acquired and congenital unilateral inner ear deafness (Baha CROS): clinical evaluation of 56 cases. *Ann Otol Rhinol Laryngol*. **119**: 447–454.
Kitterick, P.T. (2013). Cochlear implantation in patients with single-sided deafness. In: ISRCTN Regist. http://www.isrctn.com/ISRCTN33301739. Accessed 10 Aug 2020.
Kitterick, P.T. (2013). Prospective within-subject comparison of the benefits of cochlear implantation versus contralateral routing of signal (CROS) hearing aids in adult patients with single-sided deafness. In: Cochrane Cent. Regist. Control. Trials. https://www.cochranelibrary.com/central/doi/10.1002/central/CN-00887080/full. Accessed 10 Aug 2020.
Kitterick, P.T. (2015). Cochlear implantation in patients with single-sided deafness. In: ISRCTN Regist. http://www.isrctn.com/ISRCTN33301739. Accessed 10 Aug 2020.
Kitterick, P.T., O'Donoghue, G.M., Edmondson-Jones, M., Marshall, A., Jeffs, E., Craddock, L., Riley, A., Green, K., O'Driscoll, M., Jiang, D., Nunn, T., Saeed, S., Aleksy, W., & Seeber, B.U. (2014). Comparison of the benefits of cochlear implantation versus contra-lateral routing of signal hearing aids in adult patients with single-sided deafness: Study protocol for a prospective within-subject longitudinal trial. *BMC Ear Nose Throat Disord.* https://doi.org/10.1186/1472-6815-14-7.
Lin, L.-M., Bowditch, S., Anderson, M.J., May, B., Cox, K.M., & Niparko, J.K. (2006). Amplification in the rehabilitation of unilateral deafness: speech in noise and directional hearing effects with bone-anchored hearing and contralateral routing of signal amplification. *Otol Neurotol*. **27**: 172–182.
Louza, J., Hempel, J.M., Krause, E., Berghaus, A., Müller, J., & Braun, T. (2017). Patient benefit from cochlear implantation in single-sided deafness: a 1-year follow-up. *Eur Arch Oto-Rhino-Laryngology*. **274**: 2405–2409.
Lundborg, T., Swärd, I., & Lindström, B. (1976). Experience with classic CROS hearing aids in unilateral deafness. *Scand Audiol*. **5**: 17–23.
Medtronic Surgical Technologies (2017). Ambispective Clinical Evaluation of Sophono™ (ACES). In: ClinicalTrials.gov. https://clinicaltrials.gov/ct2/show/NCT03143257?cond=NCT03143257&rank=1. Accessed 10 Aug 2020.
Mertens, G., Hofkens, A., Punte, A.K., De Bodt, M., & Van De Heyning, P. (2015). Hearing performance in single-sided deaf cochlear implant users after upgrade to a single-unit speech processor. *Otol Neurotol*. **36**: 51–60.
Murray, M. (2009). Bone conduction auditory performance via the tooth for single-sided deafness. In: ClinicalTrials.gov. https://clinicaltrials.gov/ct2/show/NCT00977314?cond=NCT00977314&rank=1. Accessed 10 Aug 2020.

Murray, M. (2010). Long term safety of the Sonitus SoundBite system. In: ClinicalTrials.gov. https://clinicaltrials.gov/ct2/show/NCT01108406. Accessed 10 Aug 2020.
Nevoux, J., Coudert, C., Boulet, M., Czajka, C., Tavernier, L., Daval, M., Ayache, D., Meller, R., Rossetto, S., Papon, J.F., & Deveze, A. (2018). Transcutaneous Baha Attract system: Long-term outcomes of the French multicenter study. *Clin Otolaryngol*. **43**: 1553–1559.
Popelka, G.R. (2011). SoundBite hearing system long term multi site patient use study. In: ClinicalTrials.gov. https://clinicaltrials.gov/ct2/show/record/NCT01445977. Accessed 10 Aug 2020.
Sladen, D.P., Carlson, M.L., Dowling, B.P., Olund, A.P., Teece, K., DeJong, M.D., Breneman, A., Peterson, A., Beatty, C.W., Neff, B.A., & Driscoll, C.L. (2017). Early outcomes after cochlear implantation for adults and children with unilateral hearing loss. *Laryngoscope*. **127**: 1683–1688.
Sladen, D.P., Frisch, C.D., Carlson, M.L., Driscoll, C.L.W., Torres, J.H., & Zeitler, D.M. (2017). Cochlear implantation for single-sided deafness: A multicenter study. *Laryngoscope*. **127**: 223–228.
Smith, M.D., & Knappett, R. (2015). Hearing handicap in patients with single sided deafness. In: ClinicalTrials.gov. https://clinicaltrials.gov/ct2/show/NCT02525640. Accessed 10 Aug 2020.
Snapp, H.A., Fabry, D.A., Telischi, F.F., Arheart, K.L., & Angeli, S.I. (2010). A clinical protocol for predicting outcomes with an implantable prosthetic device (Baha) in patients with single-sided deafness. *J Am Acad Audiol*. **21**: 654–662.
Van de Heyning, P., Vermeire, K., Diebl, M., Nopp, P., Anderson, I., & De Ridder, D. (2008). Incapacitating unilateral tinnitus in single-sided deafness treated by cochlear implantation. *Ann Otol Rhinol Laryngol*. **117**: 645–652.
Wesarg, T., Aschendorff, A., Laszig, R., Beck, R., Schild, C., Hassepass, F., Kroeger, S., Hocke, T., & Arndt, S. (2013). Comparison of speech discrimination in noise and directional hearing with 2 different sound processors of a bone-anchored hearing system in adults with unilateral severe or profound sensorineural hearing loss. *Otol Neurotol*. **34**: 1064–1070.
Yang, J., Wang, Z., Huang, M., Chai, Y., Jia, H., Wu, Y., Dai, Y., Li, Y., & Wu, H. (2018). BoneBridge implantation in patients with single-sided deafness resulting from vestibular schwannoma resection: objective and subjective benefit evaluations. *Acta Otolaryngol*. **138**: 877–885.

Systematic review records (n=12) yielded by the searches were conducted and were manually hand-searched for any overlooked studies for inclusion:

Blasco, M.A., & Redleaf, M.I. (2014). Cochlear implantation in unilateral sudden deafness improves tinnitus and speech comprehension: meta-analysis and systematic review. *Otol Neurotol*. **35**: 1426–1432.
Cabral Junior, F., Hausen Pinna, M., Dourado Alves, R., dos Santos Malerbi, A.F., & Ferreira Bento, R. (2016). Cochlear implantation and single-sided deafness: A systematic review of the literature. *Int Arch Otorhinolaryngol*. **20**: 69–75.
Cohen, S.M., & Svirsky, M.A. (2019). Duration of unilateral auditory deprivation is associated with reduced speech perception after cochlear implantation: A single-sided deafness study. *Cochlear Implants Int*. **20**: 51–56.
Kim, G., Ju, H.M., Lee, S.H., Kim, H.-S., Kwon, J.A., & Seo, Y.J. (2017). Efficacy of bone-anchored hearing aids in single-sided deafness: A systematic review. *Otol Neurotol*. **38**: 473–483.
Kitterick, P.T., Lucas, L., & Smith, S.N. (2015). Improving health-related quality of life in single-sided deafness: A systematic review and meta-analysis. *Audiol Neurotol*. **20**: 79–86.
Kitterick, P.T., Smith, S.N., & Lucas, L. (2016). Hearing instruments for unilateral severe-to-profound sensorineural hearing loss in adults: a systematic review and meta-analysis. *Ear Hear*. **37**: 495–507.
Magele, A., Schoerg, P., Stanek, B., Gradl, B., & Sprinzl, G.M. (2019). Active transcutaneous bone conduction hearing implants: Systematic review and meta-analysis. *PLoS One*. **14**: e0221484.
Peter, N., Liyanage, N., Pfiffner, F., Huber, A., & Kleinjung, T. (2019). The influence of cochlear implantation on tinnitus in patients with single-sided deafness: A systematic review. *Otolaryngol - Head Neck Surg*. **161**: 576–588.
Peters, J.P.M., Smit, A.L., Stegeman, I., & Grolman, W. (2015). Review: Bone conduction devices and contralateral routing of sound systems in single-sided deafness. *Laryngoscope*. **125**: 218–226.
Sprinzl, G.M., & Wolf-Magele, A. (2016). The Bonebridge bone conduction hearing implant: Indication criteria, surgery and a systematic review of the literature. *Clin Otolaryngol*. **41**: 131–143.
van Zon, A., Peters, J.P.M., Stegeman, I., Smit, A.L., & Grolman, W. (2015). Cochlear implantation for patients with single-sided deafness or asymmetrical hearing loss: a systematic review of the evidence. *Otol Neurotol*. **36**: 209–219.
Wendrich, A.W., Kroese, T.E., Peters, J.P.M., Cattani, G., & Grolman, W. (2017). Systematic review on the trial period for bone conduction devices in single-sided deafness: Rates and reasons for rejection. *Otol Neurotol*. **38**: 632–641.
