# Supplementary material for: Systematic review of outcome domains and instruments used in designs of clinical trials for interventions that seek to restore bilateral and binaural hearing in adults with unilateral severe to profound sensorineural hearing loss (‘single-sided deafness’)
Source: Trials. 2021 Mar 20;22:220. doi: 10.1186/s13063-021-05160-5 (PMC7981927; doi:10.1186/s13063-021-05160-5)
Supplement: Supplementary file 8 — Additional file 8. Author queries. Table of records containing missing data that was queried to the corresponding author by email, and outcome. [file 13063_2021_5160_MOESM8_ESM.pdf]

Towards a Consensus on Outcome Measures for Interventions that Seek to Restore Bilateral and Binaural Hearing in Adults with Unilateral Severe-to-Profound Hearing Loss: The CROSSSD (Core Rehabilitation Outcome Set for Single Sided Deafness) Study

Table of records containing missing data that was queried to the corresponding author by email, and outcome:

Reference for the study record	Query	Outcome	Decision
Buechner, A., Brendel, M., Lesinski-Schiedat, A., Wenzel, G., Frohne-Buechner, C., Jaeger, B., & Lenarz, T. (2010). Cochlear implantation in unilateral deaf subjects associated with ipsilateral tinnitus. *Otol Neurotol*. **31**(*9*): 1381–1385.	Exact audiometric thresholds	No response	Included for sensitivity analysis
Cabral Junior, F., Hausen Pinna, M., Dourado Alves, R., dos Santos Malerbi, A.F., & Ferreira Bento, R. (2016). Cochlear implantation and single-sided deafness: A systematic review of the literature. *Int Arch Otorhinolaryngol*. **20**(*1*): 69–75.	SSD definition not explicitly defined	No response	Included for sensitivity analysis
Brendel, M., & Hamacher, V. (2018). Influence of Contralateral Routing of Signals (CROS) on hearing abilities of different groups of cochlear implant user. In: drks.de. http://www.drks.de/DRKS00013973. Accessed 16 May 2020.	Unsure if inclusion criteria allow SSD participant recruitment	Response from authors: No SSD participants included	Excluded from data extraction
Syms, C. (2013). Evaluation of benefit for treatment of Single Sided Deafness (SSD) between two bone conduction prosthetic devices; osseointegrated implant versus maxilla anchored removable oral appliance (“SoundBite”). In: ClinicalTrials.gov. https://clinicaltrials.gov/ct2/show/NCT01933386. Accessed 16 May 2020.	SSD not explicitly defined	Email to authors undeliverable	Included for data extraction as intention is to recruit SSD participants
Smith, M.D., & Knappett, R. (2015). Hearing handicap in patients with single sided deafness. In: ClinicalTrials.gov. https://clinicaltrials.gov/ct2/show/NCT02525640. Accessed 16 May 2020.	SSD criteria not explicitly defined	Response from authors: Some participants had asymmetric hearing loss	Included for sensitivity analysis
Gnansia, D., & Frachet, B. (2016). Tinnitus treatment with cochlear implant in single sided deafness. In: ClinicalTrials.gov. https://clinicaltrials.gov/ct2/show/NCT02966366. Accessed 16 May 2020.	Audiometric criteria for ‘normal or near normal’ ear not explicitly stated	Response from authors: ‘no exact criteria set for normal ear’	Included for sensitivity analysis

Willeboer, K. (2005). Parametric cochlear implant map adjustment by implant recipients. In: trialregister.nl. https://www.trialregister.nl/trial/452. Accessed 16 May 2020.	Audiometric criteria not listed	Response from authors: Study include participants with bilateral hearing loss	Excluded from data extraction
Dillon, M.T., Buss, E., Anderson, M.L., King, E.R., Deres, E.J., Buchman, C.A., Brown, K.D., & Pillsbury, H.C. (2017). Cochlear implantation in cases of unilateral hearing loss: Initial localization abilities. *Ear Hear*. **38**: 611–619.	Audiometric thresholds listed for up to 2kHz, no information on 4kHz	No response	Included for sensitivity analysis
Dillon, M.T., Buss, E., Rooth, M.A., King, E.R., Deres, E.J., Buchman, C.A., Pillsbury, H.C., & Brown, K.D. (2018). Effect of cochlear implantation on quality of life in adults with unilateral hearing loss. *Audiol Neurotol*. **22**(*4–5*): 259–271.	Not all participants meet SSD definition	No response	Included for sensitivity analysis
Doobe, G., Ernst, A., Ramalingam, R., Mittmann, P., & Todt, I. (2015). Simultaneous labyrinthectomy and cochlear implantation for patients with single-sided Ménière's Disease and profound sensorineural hearing loss. *Biomed Res Int*. https://doi.org/10.1155/2015/457318.	Hearing thresholds for non-Ménière's disease ear not explicitly listed	First author responded; team were to follow up, no subsequent response	Included for sensitivity analysis
Dumon, T., Medina, M., & Sperling, N.M. (2016). Punch and Drill: Implantation of bone anchored hearing device through a minimal skin punch incision versus implantation with dermatome and soft tissue reduction. *Ann Otol Rhinol Laryngol*. **125**: 199–206.	Insufficient details given to check if participants met PICO. Just states "…candidacy based on clinical parameters" without stating what these were	Response from authors: Still unclear, asked for further clarification, no response	Excluded from data extraction
Hill, S.L. 3rd, Marcus, A., Digges, E.N.B., Gillman, N., & Silverstein, H. (2006). Assessment of patient satisfaction with various configurations of digital CROS and BiCROS hearing aids. *Ear Nose Throat J*. **85**(*7*): 427–430.	Exact audiometric thresholds for 'asymmetric hearing loss' not stated	Email to authors undeliverable	Excluded from data extraction
Hol, M.K.S., Kunst, S.J.W., Snik, A.F.M., & Cremers, C.W.R.J. (2010). Pilot study on the effectiveness of the conventional CROS, the transcranial CROS and the BAHA transcranial CROS in adults with unilateral inner ear deafness. *Eur Arch Otorhinolaryngol*. **267**(*6*): 889–896.	Audiometric thresholds of poor ear not explicitly listed	No response	Included for sensitivity analysis
Kubo, T., Yamamoto, K.I., Iwaki, T., Doi, K., & Tamura, M. (2001). Different forms of dizziness occurring after cochlear implant. *Eur Arch Oto-Rhino-Laryngology*. **258**(*1*): 9–12.	Audiometric criteria not listed	Email to authors undeliverable	Excluded from data extraction
Louza, J., Hempel, J.M., Krause, E., Berghaus, A., Müller, J., & Braun, T. (2017). Patient benefit from cochlear implantation in	Audiometric thresholds for poor ear not explicitly listed	No response	Included for sensitivity analysis

single-sided deafness: a 1-year follow-up. *Eur Arch Oto-Rhino-Laryngology*. **274**(*6*): 2405–2409.			
Sladen, D.P., Carlson, M.L., Dowling, B.P., Olund, A.P., Teece, K., DeJong, M.D., Breneman, A., Peterson, A., Beatty, C.W., Neff, B.A., & Driscoll, C.L. (2017). Early outcomes after cochlear implantation for adults and children with unilateral hearing loss. *Laryngoscope*. **127**(*7*): 1683–1688.	Audiometric criteria for 'unilaterally deaf' not explicitly stated	Response from authors: A few participants do not fit the SSD definition in worse ear	Included for sensitivity analysis
Song, J.-J., Punte, A.K., De Ridder, D., Vanneste, S., & Van de Heyning, P. (2013). Neural substrates predicting improvement of tinnitus after cochlear implantation in patients with single-sided deafness. *Hear Res*. **299**: 1–9.	SSD criteria not explicitly defined	Response from authors: All participants fit SSD definition	Included for data extraction and synthesis
