# Supplementary material for: Systematic review of outcome domains and instruments used in designs of clinical trials for interventions that seek to restore bilateral and binaural hearing in adults with unilateral severe to profound sensorineural hearing loss (‘single-sided deafness’)
Source: Trials. 2021 Mar 20;22:220. doi: 10.1186/s13063-021-05160-5 (PMC7981927; doi:10.1186/s13063-021-05160-5)
Supplement: Supplementary file 9 — Additional file 9. Complete list of reported outcome domains. A comprehensive list of all reported primary and secondary outcome domains reported across the 72 included studies, classified according to the Dodd et al. [90] taxonomy. Categories within each core area are arranged by the most frequently used first. The number of individual studies that reported each primary and secondary domain is also listed. N/A = none reported. [file 13063_2021_5160_MOESM9_ESM.pdf]

Additional file 09. A comprehensive list of all reported primary and secondary outcome domains, classified according to the Dodd *et al.* (2018) taxonomy. Categories within each core area are arranged by the most frequently used first. The number of individual studies that reported each primary and secondary domain is also listed. - = none reported.

**Taxonomy core area**	**Taxonomy categories** (number of studies reporting as primary / secondary outcome)	**Outcome domain**	**Number of times primary outcome domain reported** (number of studies reporting as primary outcome)	**Number of times (n) secondary outcome domain reported** (number of studies reporting as secondary outcome)
Physiological or Clinical	6: Ear and labyrinth outcomes (194, 90)	Speech in noise	64 (45)	15 (14)
		Localisation	36 (29)	5 (4)
		Speech in quiet	21 (17)	2 (2)
		Hearing thresholds	20 (13)	-
		Speech hearing	13 (12)	21 (16)
		Tinnitus loudness	9 (7)	4 (2)
		Spatial hearing	6 (6)	16 (8)
		Quality of hearing	5 (2)	9 (7)
		Reverberation	5 (5)	10 (10)
		Binaural hearing	5 (2)	-
		Psychoacoustic performance	5 (3)	-
		Motion perception	2 (1)	-
		Hyperacusis	1 (1)	3 (1)
		Loudness of sound	-	1 (1)
		Middle ear	1 (1)	-
		Softness of sound	-	1 (1)
		Tinnitus perception	1 (1)	2 (1)
		Tinnitus-related hearing	-	1 (1)
	7: Eye outcomes (0, 1)	Vision	-	1 (1)
	9: General outcomes (9, 1)	Dental	8 (1)	-
		Pain	1 (1)	1 (1)
	17: Nervous system outcomes (3, 3)	Brain activity	2 (2)	3 (3)
		Brain activity (tinnitus related)	1 (1)	-
	21: Psychiatric outcomes (3, 0)	Mental health	3 (2)	-

	23: Skin and subcutaneous tissue outcomes (2, 0)	Skin safety	2 (1)	-
Life Impact	25: Physical functioning (2, 5)	Ambulation	-	1 (1)
		Dexterity	-	1 (1)
		Physical health	1 (10	2 (2)
		Tinnitus-related physical problems	-	1 (1)
		Vitality	1 (1)	-
	26: Social functioning (1, 3)	Participation restrictions	-	1 (1)
		Social support	-	2 (2)
		Social impact	1 (1)	-
	27: Role functioning (1, 1)	Activity limitations	1 (1)	1 (1)
	28. Emotional functioning (7, 5)	Tinnitus annoyance	2 (1)	-
		Tinnitus-related distress	2 (1)	2 (2)
		Coping	1 (1)	-
		Stress	1 (1)	-
		Work-related stress	1 (1)	-
		Emotion	-	1 (1)
		Tinnitus intrusiveness	-	1 (1)
		Tinnitus-related sleep problems	-	1 (1)
	29: Cognitive functioning (2, 3)	Listening effort	2 (2)	1 (1)
		Cognition	-	1 (1)
		Tinnitus-related cognition	-	1 (1)
	30: Global quality of life (55, 16)	Tinnitus symptom severity	22 (15)	2 (2)
		Hearing disability	14 (14)	2 (2)
		Disease-specific quality of life	9 (9)	8 (6)
		Health-related quality of life	4 (4)	2 (1)
		Dizziness	3 (1)	-
		Hearing handicap	2 (2)	1 (1)
		Pre-intervention disability	1 (1)	1 (1)
	31: Perceived health status (1,0)	General health	1 (1)	-
	32a: Delivery of care - Satisfaction / patient preference (51, 17)	Device benefit	19 (16)	1 (1)
		Device use	11 (11)	1 (1)
		Satisfaction	8 (6)	3 (2)
		Aversiveness	5 (5)	6 (6)
		Residual (aided) disability	2 (2)	1 (1)
		Clarity of sound	1 (1)	1 (1)
		Device performance	1 (1)	-

		Device preference	2 (2)	-
		Likelihood of recommending	1 (1)	-
		Work-related performance	1 (1)	-
		Brightness of sound	-	1 (1)
		Fullness of sound	-	1 (1)
		Hearing benefit	-	1 (1)
	32b: Delivery of care - Acceptability and availability (0, 1)	Self-image and stigma	-	1 (1)
Resource Use	34: Economic (2, 2)	Cost	1 (1)	2 (2)
		Productivity loss	1 (1)	-
	35: Hospital (1, 0)	In-patient stay	1 (1)	-
	37: Societal / carer burden (0, 1)	Impact on others	-	1 (1)
Adverse Events	38: Adverse events / effects (11, 5)	Adverse effects	7 (6)	5 (4)
		Safety	3 (1)	-
		Device failure	1 (1)	-
	0: Cannot Code (5,17)	Not stated	5	17
